# The NSL complex-mediated nucleosome landscape is required to maintain transcription fidelity and suppression of transcription noise

**DOI:** 10.1101/gad.321489.118

**Published:** 2019-04-01

**Authors:** Kin Chung Lam, Ho-Ryun Chung, Giuseppe Semplicio, Shantanu S. Iyer, Aline Gaub, Vivek Bhardwaj, Herbert Holz, Plamen Georgiev, Asifa Akhtar

**Affiliations:** 1 Department of Chromatin Regulation, Max Planck Institute of Immunobiology and Epigenetics, 79108 Freiburg, Germany;; 2 Otto-Warburg-Laboratory, Epigenomics, Max Planck Institute for Molecular Genetics, 14195 Berlin, Germany;; 3 Institute for Medical Bioinformatics and Biostatistics, Philipps-Universität Marburg, 35037 Marburg, Germany;; 4 Spemann Graduate School of Biology and Medicine (SGBM), University of Freiburg, 79108 Freiburg, Germany;; 5 Faculty of Biology, University of Freiburg, 79108 Freiburg, Germany

**Keywords:** NDR, NSL, NURF, nucleosome, transcription

## Abstract

Nucleosomal organization at gene promoters is critical for transcription, with a nucleosome-depleted region (NDR) at transcription start sites (TSSs) being required for transcription initiation. How NDRs and the precise positioning of the +1 nucleosomes are maintained on active genes remains unclear. Here, we report that the *Drosophila* nonspecific lethal (NSL) complex is necessary to maintain this stereotypical nucleosomal organization at promoters. Upon NSL1 depletion, nucleosomes invade the NDRs at TSSs of NSL-bound genes. NSL complex member NSL3 binds to TATA-less promoters in a sequence-dependent manner. The NSL complex interacts with the NURF chromatin remodeling complex and is necessary and sufficient to recruit NURF to target promoters. Not only is the NSL complex essential for transcription, but it is required for accurate TSS selection for genes with multiple TSSs. Furthermore, loss of the NSL complex leads to an increase in transcriptional noise. Thus, the NSL complex establishes a canonical nucleosomal organization that enables transcription and determines TSS fidelity.

## Abstract (precis)

In this study, Lam et al. report that the *Drosophila* nonspecific lethal (NSL) complex is necessary to maintain positioning of nucleosomal organization at gene promoters. Their findings show that the NSL complex establishes a canonical nucleosomal organization that enables transcription and determines TSS fidelity.

Chromatin structure and organization are fundamental to the regulation of gene transcription. Chromatin at active gene promoters is characterized by a distinct nucleosomal organization (Lai and Pugh 2017). Transcription start sites (TSSs) are embedded in a nucleosome-depleted region (NDR), which enables preinitiation complex formation (Workman and Roeder 1987; Dutta and Workman 2012). The NDR is bordered by the well-positioned +1 nucleosome followed by a regular array of nucleosomes. This organization is thought to be required for transcription initiation (Lai and Pugh 2017).

The standard model of transcription initiation is based on genes that are activated in a tissue-specific manner (Kadonaga 2012). The current model supports that TBP binds to the TATA box at promoters of tissue-specific genes, where the assembly of RNA polymerase II (Pol II) is initiated and TFIIA, TFIIB, TFIID, TFIIE, TFIIF, and TFIIH form the preinitiation complex. TBP–TATA-box binding occurs at ∼30 bp upstream of the TSS and thus defines a sharp and precise TSS (Carninci et al. 2006; Yamamoto et al. 2009; Ni et al. 2010). However, this model does not represent the majority of genes, as multicellular organisms express a range of housekeeping genes that are critical for homeostatic maintenance. Unlike tissue-specific genes, housekeeping genes have highly dispersed TSSs that are scattered over up to 100 bp (Carninci et al. 2006; Ni et al. 2010). This difference in transcription initiation patterns between housekeeping and tissue-specific gene promoters are conserved across species including fish, flies, and mammals (Carninci et al. 2006; Ni et al. 2010; Haberle et al. 2014).

The precise selection of TSS is dependent on the tissue and developmental stage (Haberle et al. 2014) and thus pose an important aspect of transcription regulation as changes in TSS can affect RNA stability and the resulting protein isoforms. Despite its importance, the nature and the causative relationship of the DNA sequence and transcription factors that direct TSS selection at housekeeping genes remain poorly understood. Compared with focused promoters of tissue-specific genes, dispersed housekeeping gene promoters contain distinct sets of core promoter motifs and binding proteins (Vo Ngoc et al. 2017). Dispersed promoters in *Drosophila* generally lack a TATA box or Inr elements but rather contain motif 1, motif 6, motif 7, and DRE (Rach et al. 2009; Vo Ngoc et al. 2017). Although the TATA box initiates the binding of TBP and then Pol II, it is still not clear what instructs Pol II to initiate transcription at the dispersed promoters in the absence of a TATA box or Inr elements. Likewise, distinct set of proteins are found on dispersed promoter: Motif 1-binding protein (M1BP) recognizes motif 1 and DREF binds to DREs. Therefore, they are believed to be binding to the dispersed promoters only. The difference in DNA motifs, protein binding and transcription patterns between dispersed housekeeping and focused promoters argues for fundamental differences in the mechanisms of transcription initiation.

The distinction between the two major types of promoters could be a result of chromatin-modifying factors that influence the local chromatin modifications and organization. Elegant work in yeast, flies, and mammals has shown the importance of chromatin remodeling complexes in nucleosome organization (Alkhatib and Landry 2011; Struhl and Segal 2013; Lai and Pugh 2017). Chromatin remodeler complexes can be broadly classified into four families (ISWI, CHD/Mi- 2, INO80/SWR1, and SWI/SNF) based on the protein domains of their catalytic ATPase subunits. Each remodeler has its characteristic molecular structure, target genomic locations, and roles in cells. In higher eukaryotes, it is not yet clear which *trans*-acting factors are responsible for the nucleosomal organization at TSSs. How chromatin remodeling complexes work in concert with other chromatin-modifying enzymes and transcription machinery to facilitate the transcription process remains an active area of research (Struhl and Segal 2013; Lai and Pugh 2017).

The *Drosophila* nonspecific-lethal (NSL) complex is a chromatin-modifying complex. It contains the histone H4 Lys16 acetyltransferase MOF, as well as NSL1, NSL2, NSL3, MCSR2, MBDR2, Z4, Chromator, and WDS (Mendjan et al. 2006; Raja et al. 2010). Underpinning its importance, loss of NSL complex members leads to lethality during early development in flies (Raja et al. 2010), whereas heterozygous mutations in NSL1 and NSL2 orthologs *KANSL1* and *KANSL2* underlie intellectual disability in humans (Koolen et al. 2012; Zollino et al. 2012; Gilissen et al. 2014). The NSL complex binds to the dispersed housekeeping gene promoters, and this feature is remarkably conserved from flies to human (Lam et al. 2012; Chelmicki et al. 2014; Ravens et al. 2014). However, the mechanism by which the NSL complex regulates housekeeping gene expression remains unknown. Therefore, studying how the NSL complex functions is an important paradigm to understand how transcription factors specifically target the vast number of housekeeping genes and mediate transcription in a way that is fundamentally different from what we typically associate with tissue-specific or developmental genes.

Here, we report that the NSL complex is necessary to maintain the stereotypical nucleosomal organization at promoters. Upon NSL1 depletion, nucleosomes invade the NDR at the TSS of NSL-bound genes. We also uncover that binding of the NSL complex to TATA-box-less housekeeping gene promoters is directed by AT-rich sequences. Accordingly, we can predict the in vivo NSL complex binding by AT-rich sequences and chromatin context. Mechanistically, we show that the NSL complex recruits the NURF complex to maintain the nucleosome pattern that is typical of dispersed promoters. This nucleosome pattern is important for gene regulation as its disruption leads to spurious TSS selection and an increase in transcriptional noise. Our data illustrate how housekeeping gene promoters can be targeted by the NSL complex, which then impose a specific nucleosome pattern, TSS selection, and transcription noise regulation in the *Drosophila* genome.

## Results

### NSL complex loss leads to reduced nucleosome patterning at target TSSs

**Figure 1.**
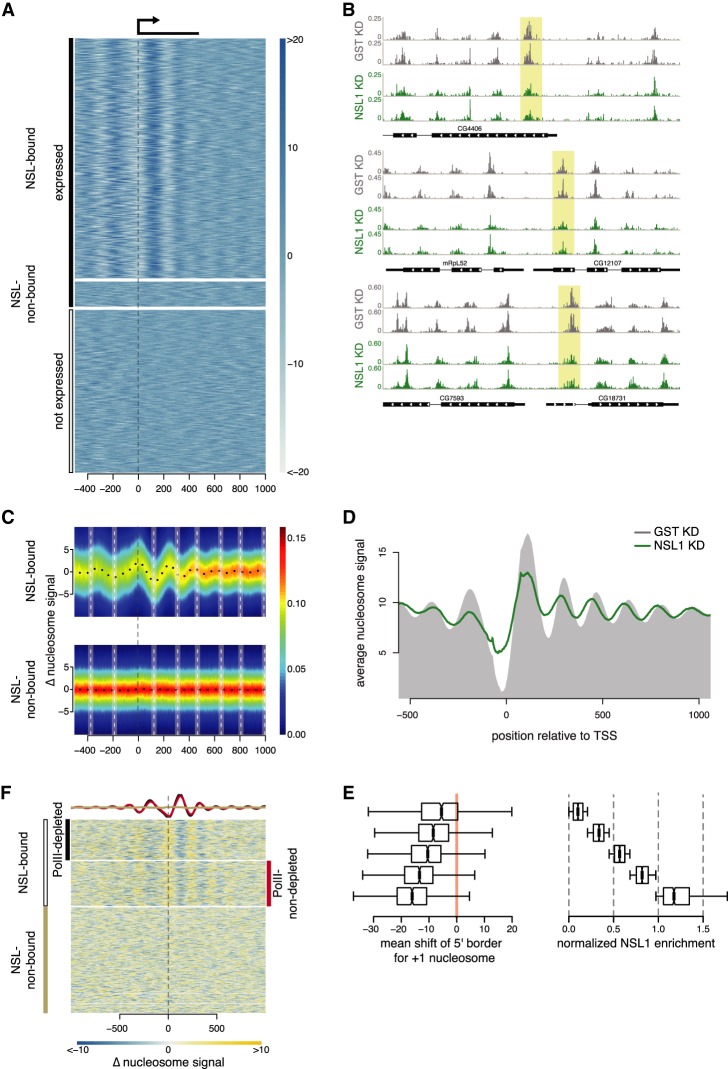
NSL complex loss leads to reduced nucleosome patterning at target TSSs. (*A*) Heat map showing the nucleosome signal (see Materials and Methods) on NSL-bound active genes (*top*), NSL-nonbound active genes (*middle*), and inactive genes (*bottom*), −500 to +1000 bp of the TSS. Active genes had RNA sequencing (RNA-seq) reads covering their exons, whereas inactive genes had none. (*B*) Three representative examples of nucleosome density (read counts normalized to sequencing depth using RPGC) in control (gray) and NSL1 knockdown (green). The +1 nucleosomes are shaded in yellow. (*C*) Heat map depicting the difference in nucleosome densities in control and NSL1 knockdown −500 bp to +1000 bp of the TSS of NSL-bound genes (*top*) and NSL-nonbound (*bottom*) genes. The *y*-axis represents changes in nucleosome signal. The white vertical dashed lines denote wild-type nucleosome positions. The color scale bar indicates scatter density. (*D*) Summary plot showing the nucleosome positions in wild-type (gray area) and NSL1-depleted (green line) cells. (*E*) Quantification of the shift of the +1 nucleosomes in base pairs (*left*) for five groups of MapCap TSSs (6281) with increasing (*top* to *bottom*) NSL1 log_2_ chromatin immunoprecipitation (CHIP)/input ratio (*right*). (*F*) Heat map showing changes in nucleosome density upon NSL1 knockdown for NSL-bound genes with Pol II loss upon NSL1 knockdown (*top*), NSL-bound genes with no Pol II loss (*middle*), and NSL-nonbound genes (*bottom*). The three groups were determined by first kmean clustering the promoters by the summed log_2_ enrichment of NSL3 and MBDR2 in a window ±290 bp around the annotated TSS into NSL-bound and -nonbound. The promoters of the NSL-bound genes were further subdivided into promoters that loose Pol II and that retain Pol II by kmeans clustering by the average difference of Pol II log_2_ enrichment between control and NSL1 knockdown in a window ±145 bp around the annotated TSS. The summary plot depicts the average nucleosome density of NSL-bound genes with Pol II loss (black) and NSL-bound genes without Pol II loss (red).

Because the NSL complex is an important regulator for the majority of active promoters (Lam et al. 2012; Chelmicki et al. 2014), we sought to understand its roles in establishing the chromatin landscape at promoters. To study if the NSL complex is required for nucleosomal organization, we knocked down NSL1 and GST (control) in *Drosophila* S2 embryonic cells and performed micrococcal nuclease digestion followed by high-throughput sequencing (MNase-seq) (Fig. 1A; Supplemental Fig. S1A–C). Many genes showed a decrease in the nucleosome signal at +1 nucleosome position upon NSL depletion, mostly pronounced at promoter regions (Fig. 1B,C). Nucleosome occupancy changed around NSL-bound, but not around NSL-nonbound, promoters (Fig. 1C). We observe a decrease in the occupancy of the +1 nucleosome, concomitant with an increased occupancy at the NDR, indicating an invasion of the +1 nucleosome into the NDR. This was supported by nucleosome profiles in control and NSL1 knockdown, showing an average shift of the +1 nucleosome toward the TSS (Fig. 1D). Downstream from the +1 nucleosome, the array also shifts toward the TSS. The mean 5′ end position of the +1 nucleosome shifts upstream toward the TSS in a NSL1 binding-dependent manner (Fig. 1E).

The NSL complex is required for the recruitment of RNA Pol II (Lam et al. 2012); thus, changes in the nucleosomal organization could be a secondary consequence of the loss of Pol II. To address this issue, we categorized genes into three groups: (1) NSL-bound with Pol II loss, (2) NSL-bound without Pol II loss upon NSL1 knockdown, and (3) genes not targeted by NSL. The changes in the nucleosomal organization correlated with NSL binding (groups 1 and 2) and were present irrespective of Pol II loss. Group 3 genes failed to show any major changes (Fig. 1F). The subclassification of NSL targets that lose or do not lose Pol II does not depend on the wild-type Pol II levels (Supplemental Fig. S1D). Thus, the NSL complex affects the nucleosomal organization at promoters independent of the changes in Pol II recruitment.

### The NSL complex recruits the NURF complex and maintains nucleosome pattern at promoters

**Figure 2.**
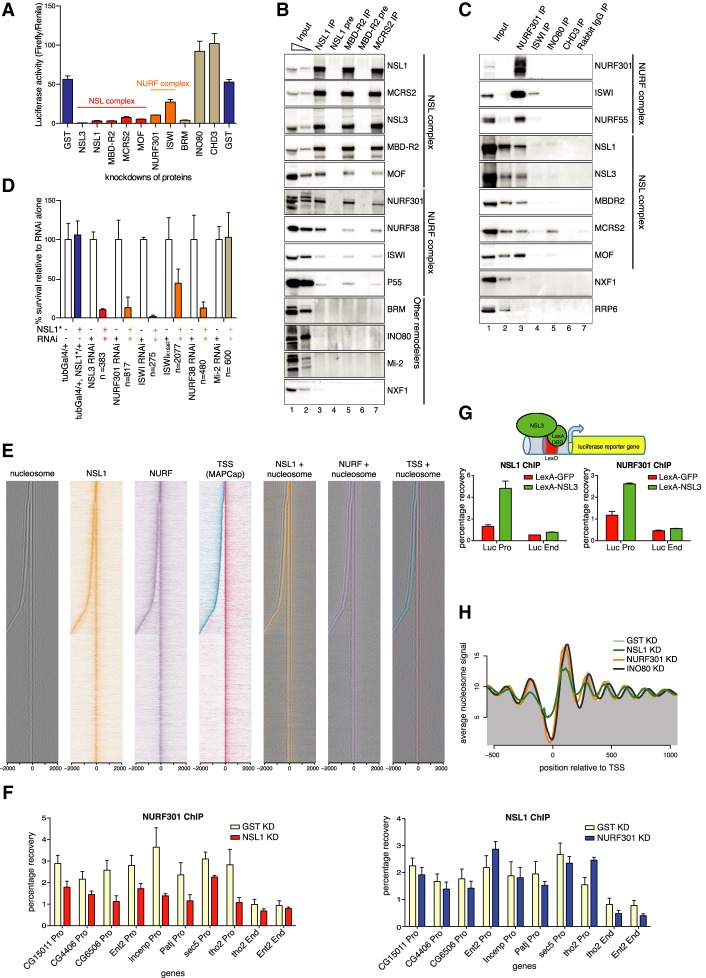
The NSL complex recruits the NURF complex and maintains nucleosome pattern at promoters. (*A*) Luciferase activity (ratio of Firefly luciferase/Renila luciferase) in a Gal4-NSL3 reporter system in knockdowns for GST (control, far *left* and *right*), NSL and NURF complex members, BRM, INO80, and CHD3. Error bars, SD of three independent experiments. (*B*) Immunoprecipitation of the endogenous NSL complex members NSL1, MBDR2, and MCRS2. Preimmune Sera (NSL1, MBD-R2) served as controls. The western blot was probed by antibodies against the NSL and NURF complexes, other chromatin remodelers, and NXF1, a protein involved in mRNA export (as a negative control). (*C*) Immunoprecipitation of the chromatin remodelers NURF301, ISWI, INO80, and CHD3. Rabbit IgG served as a control. The western blot was probed by antibodies against the NSL and NURF complexes. INO80 immunoprecipitates MCRS2, but not other NSL complex members. NXF1 and RRP6 served as negative controls. (*D*) Bar chart showing of viability of flies when combining knockdown of NSL3, NURF301, ISWI (dominant-negative mutant, marked with asterisk, and RNAi), NURF38, or Mi-2 with heterozygous NSL1 mutant (blue bars) relative to RNAi/mutant alone (white bars). Heterozygous NSL1 mutant alone (blue bar) does not cause lethality. The mean ± SD of at least three independent crosses is shown. The numbers of flies counted are indicated by *n*. (*E*) Heat maps displaying the input-normalized ChIP enrichments of NSL1 (orange) and NURF301 (purple) ±2 kb around the MAPCap TSS. Nucleosome signal is depicted in black. MAPCap TSS positions are indicated in red (Watson strand) and blue (Crick strand). (*Right*) Nucleosome signals are overlaid with the ChIP combined with high-throughput sequencing (ChIP-seq) and TSS signal. (*F*) ChIP-qPCR analyses for NURF301 (*top*) and NSL1 (*bottom*) for knockdowns against GST (pale yellow), NSL1 (red), and NURF301 (blue). qPCR analysis was performed with primer sets positioned at the promoters (Pro) and ends (End) of indicated genes. Results are expressed as mean (±SD) of relative percentage recovery of immunoprecipitated material over input material. (*G*) Schematic: LexA-NSL3 is used to activate expression of lexO-luciferase reporter in transgenic flies. Bar charts: ChIP-qPCR experiments performed with NSL1 (*left*) and NURF301 (*right*) antibodies and primer pairs specific for promoter and end regions of the reporter gene. Results are expressed as mean (±SD) of the relative percentage recovery of immunoprecipitated material over input material. (*H*) Summary plot depicting the nucleosome signal −500 to 1000 bp of the TSS in wild-type (gray shaded area), NSL1-depleted (green), NURF301-depleted (orange), and INO80-depleted (purple) cells.

As NSL complex members have no chromatin remodeling activity reported to date, we asked whether they function with chromatin remodelers to position nucleosomes at promoters (Supplemental Fig. S2A). We used a Gal4-NSL3 reporter system (Raja et al. 2010; Lam et al. 2012), in which tethering of Gal4-NSL3 to the promoter of a UAS-driven luciferase reporter results in luciferase up-regulation in S2 cells (Fig. 2A). We performed RNAi against a candidate set of chromatin remodelers (NURF301, ISWI, BRM, INO80, and CHD3) (Supplemental Fig. S2B). Knockdown of NURF301 caused a strong reduction in luciferase activity, comparable with the reduction observed upon MOF knockdown (Fig. 2A). Knockdown of ISWI led to a milder decrease. Knockdown of BRM caused a strong decrease in luciferase activity. However, the protein level of MOF was severely reduced upon BRM knockdown, which was not true in NURF301 and ISWI knockdowns (Supplemental Fig. S2B). In contrast, INO80 and CHD3 knockdowns did not attenuate NSL3-mediated activation. Thus, the NURF complex is required for NSL3-mediated transcription activation.

To determine whether the NSL complex physically interacts with the NURF complex, we immunoprecipitated endogenous NSL complex members (NSL1, MBD-R2, and MCRS2) from nuclear S2 cell extracts using polyclonal antibodies. Immunoprecipitation experiments successfully enriched for the respective NSL proteins, the NSL complex members (NSL1, NSL3, MCRS2, MBD-R2, and MOF) (Fig. 2B), and all four members of the NURF complex (NURF301, ISWI, NURF38, and p55) (Alkhatib and Landry 2011), albeit substoichiometrically (Fig. 2B). To validate these results, we performed immunoprecipitation experiments with an anti-Flag antibody in cell lines expressing NSL2-Flag or MBD-R2-Flag, both of which coimmunoprecipitated endogenous NSL complex members as well as the endogenous NURF complex members (NURF301, NURF38, and ISWI), but not INO80 (Supplemental Fig. S2C). We could specifically immunoprecipitate endogenous NSL proteins using antibodies raised against NURF301 but not against INO80 and CHD3 (Fig. 2C). Although ISWI antibody immunoprecipitated ISWI protein, it failed to enrich other NURF complex members. Thus, it was difficult to reach a conclusive interpretation from the ISWI immunoprecipitation. Immunoprecipitation of the chromatin remodeler INO80 exclusively pulls down MCRS2 (Fig. 2C). Homologs of MCRS2 and INO80 have been reported to form a complex in mammals (Cai et al. 2006; Chen et al. 2011), which is distinct from the MCRS2–NSL complex (Cai et al. 2010). Consistently, the recombinant NURF complex interacts in in vitro pulldown assays with full-length recombinant NSL1 but not with MCRS2 or MSL3 (Supplemental Fig. S2D). Thus, the NSL complex biochemically interacts with the NURF complex.

To address the relevance of these findings in vivo, we assayed the genetic interactions between *nsl1* and the *Nurf301*, *Iswi*, and *Nurf38* members of the NURF complex in flies. Loss-of-function mutants of *nsl1*, *Nsl3*, *Nurf301*, *Iswi*, *Nurf38*, and *Mi-2* are lethal. However, heterozygous *nsl1* mutants, although shown to possess reduced NSL1 activity (Yu et al. 2010), are 100% viable (Fig. 2D, blue bar). We therefore tested the ability of heterozygous *nsl1* loss-of-function alleles, either *nsl1*^*J2E5*^ (Spradling et al. 1999) or *nsl1*^*e(nos)1*^ (Yu et al. 2010), to modify (enhance or suppress) the partial silencing and lethality induced by RNAi targeting *Nsl3*, *Nurf38*, *Nurf301*, *Iswi*, and *Mi-2* (for details, see Materials and Methods). We observed a strong negative genetic interaction between *nsl1* and *Nsl3,* as expected for members of the same complex. An equally strong negative interaction was scored between *nsl1* and all tested NURF complex members, whereas no genetic interaction was seen between *nsl1* and *Mi-2* (Fig. 2D). The negative genetic interaction was equally strong in both males and females (Supplemental Fig. S2E). In further support of our genetic analysis, when a previously characterized dominant-negative *Iswi*^*K159R*^ allele (Corona et al. 1999; Deuring et al. 2000) was used in combination with a heterozygous *nsl1* mutant, a strong negative genetic interaction was again observed (Fig. 2D). These findings indicate that NSL1 and the NURF complex interact in vivo in the same or parallel converging pathways.

We sought to determine whether this interaction is required only for a specific subset of genes or is a general mechanism. Chromatin immunoprecipitation combined with high-throughput sequencing (MNase-ChIP-seq) experiments against NSL1 and NURF301 revealed that 21,138 (91%) of the 23,194 NSL1-binding sites were also bound by NURF301. Conversely, 66% of the 32,232 NURF301-binding sites were co-occupied by NSL1, and 9004 (64%) of these cobound sites overlapped with 14,081 annotated TSSs in the *Drosophila* genome, whereas only 605 (4%) and 2182 (15%) were bound by either NSL1 or NURF301, respectively (Supplemental Fig. S2F,G). Next, we used a CAGE-based approach (MAPCap) (see the Materials and Methods) to map dominant TSSs—i.e., the TSS with the most reads, at single-base-pair resolution for each gene—and sorted the genes by the distance to their closest upstream antisense TSS, within 2000 bp (Fig. 2E; Supplemental Fig. S6A). The nucleosomes aligned closely with both the sense and antisense TSS, and both the sense and antisense TSS showed extensive cobinding of NSL1 and NURF301. NSL1 and NURF301 colocalized at the NDR upstream of the +1 nucleosome, which is most affected upon NSL1 knockdown. When we overlaid the signal with TSS positions, the two proteins bind in close proximity to the TSS (Fig. 2E). Similar results were obtained when ChIP-seq data for NURF301, ISWI, ACF1, and Mi-2 were analyzed (Contrino et al. 2012; Feller et al. 2012). The NSL complex binding sites coincide extensively with the NURF complex but not ACF1 (in the ACF–ISWI complex) or Mi-2 (Supplemental Fig. S3A–C).

To understand the epistatic relationship between NSL and NURF complexes, we performed ChIP followed by qPCR assays at selected target promoters under either NSL1 or NURF301 knockdown conditions (Supplemental Fig. S2B,H,I). Knockdown of NSL1 compromised NURF301 binding to these promoters, whereas knockdown of NURF301 left NSL1 binding unchanged (Fig. 2F), indicating that the NSL complex acts upstream of NURF complex recruitment. To validate this result, we used flies carrying a lexO-luciferase reporter transgene and another transgene expressing lexA-NSL3. Tethering of NSL3 led to ectopic recruitment of NSL1, as well as NURF301 (Fig. 2G).

To determine whether recruitment of the NURF complex could explain the defects in nucleosomal organization observed upon NSL1 knockdown, we depleted NSL1, and the remodelers NURF301, and INO80 in S2 cells (Supplemental Table S1). MNase-seq experiments revealed that nucleosomes displayed a similar shift toward the TSS upon NURF301 knockdown (Fig. 2H; Supplemental Fig. S4A; Kwon et al. 2016), whereas depletion of INO80 led to a shift of nucleosomes away from the TSS. It has been reported that nucleosomes at promoters display differential sensitivity to MNase digestion in *Drosophila* (Chereji et al. 2016). To test if our results are robust in different digestion conditions, we performed our MNase-seq with various different MNase concentrations. Indeed, we can obtain the same conclusion in all digestion conditions (Supplemental Fig. S4B). Interestingly, only NSL1 knockdown led to a change in nucleosome occupancies, indicating that the NSL complex plays an additional role in maintaining the nucleosome pattern, consistent with the NSL complex being upstream of NURF complex recruitment. Thus, first, the NSL complex is important for maintaining nucleosome occupancy at +1 nucleosomes, and second, it recruits the NURF complex to position the nucleosomes at active TSSs in the *Drosophila* genome.

### The NSL complex targets TATA-less promoters by recognizing AT content

**Figure 3.**
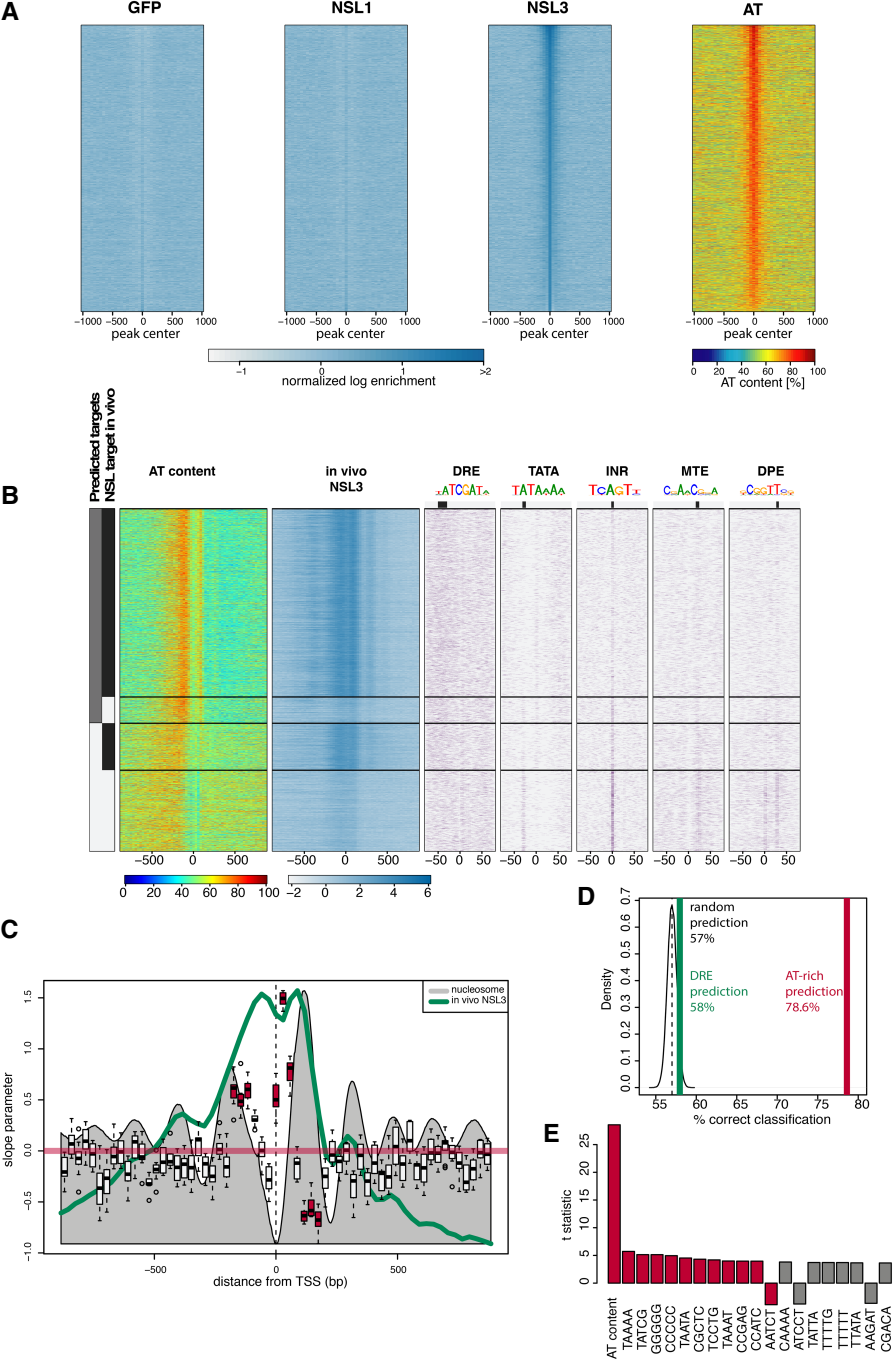
The NSL complex targets TATA-less promoters by recognizing AT content. (*A*) Heat maps showing the normalized in vitro DNA-binding signal of GFP, NSL1, and NSL3 (*left* to *right*; blue) using *Drosophila* genomic DNA. The heat maps are centered around the in vitro NSL3 binding peak center and ordered by binding intensity. AT contents of the same regions are displayed on the far *right* heat map (red). (*B*, far *left*) Bars showing predicted NSL3 binding from AT content (gray) and in vivo NSL3 binding as detected by ChIP-seq experiment (black). The first heat map depicts AT content, whereas the second heat map shows in vivo NSL3 binding. The following heat maps on the *right* show occurrence of PWM hits for core promoter motifs DRE, TATA, INR, MTE, and DPE, respectively. For each motif, a sequence logo and its preferred location are indicated. The genes are clustered into four groups along the *y*-axis: (1) genes predicted to be bound and are bound in vivo, (2) genes predicted to be bound but are not bound in vivo, (3) genes predicted not to be bound but are bound in vivo, and (4) genes predicted not to be bound and are not bound in vivo. (*C*) Box plot showing 29-bp bins that are significantly contributing to prediction of NSL3 binding. The *x-*axis denotes the position with respect to the MapCap TSSs. The *y*-axis denotes the slope parameter values obtained during the 10-fold cross validation to predict NSL3 binding. The gray filled wiggle line denotes the nucleosome signal. The green line denotes the NSL3 in vivo binding. The red horizontal line denotes a slope of zero. Red boxes denote bins that successfully predict behavior of NSL3 binding. *t*-test, *P*-value < 0.05. (*D*) In vivo NSL3 binding is predicted using random sequences (black), DRE (green), and AT-rich sequences (red) using the method described in *C*. The percentage of sequences making a correct prediction are indicated on the *x*-axis. (*E*) Bar charts showing *t*-statistics representing partial correlation of the indicated elements to in vitro binding of NSL3. The AT content (percentage against the log enrichments for all bins) and frequencies of all 1024 possible 5mers are calculated for 200-bp bins. The in vitro NSL3 log enrichment was linearly regressed against the AT content and the frequencies of all possible 5mers used to calculate the partial correlation coefficient for the 5mers.

Because the NSL complex appeared upstream of NURF recruitment, we next addressed how the NSL complex recognizes target promoters. Previous genome-wide correlations suggested association of DRE sequence with NSL target sites (Feller et al. 2012; Lam et al. 2012). However, whether this or other elements could be specifically targeted by the NSL complex remained unknown. For this purpose, we performed DNA immunoprecipitation by isolating and shearing *Drosophila* genomic DNA and incubating it with recombinant NSL1, NSL3, MCRS2, and GFP as controls (Supplemental Fig. S5A). The bound DNA was subsequently purified and sequenced. Peak calling revealed that the GFP control enriched 3726, MCRS2 enriched only six, NSL1 enriched 1910, and NSL3 enriched 4614 regions (Supplemental Fig. S5B). The achieved enrichment for MCRS2 and NSL1 remain very close to the enrichment obtained by the GFP control. Only the enrichments achieved with NSL3 are higher. Furthermore, 733 NSL1 regions overlapped with GFP regions, whereas only 29 NSL3 regions did so. Our data do not provide any positive evidence that MCRS2 binds specifically to DNA, but they do not rule out that MCRS2 could bind to DNA under a different condition as tested here. This inconclusive result promoted us to remove MCRS2 from further consideration. NSL1 regions have similar enrichments as the GFP control and overlap extensively with the GFP control, suggesting that the uncovered binding regions for these two proteins are unspecific. Again, this finding does not rule out that NSL1 binds specifically to DNA. NSL3 binds to specific regions in the genome, characterized by high AT content (Fig. 3A; Supplemental Fig. S5C). We observed that the AT-rich sequences overlap extensively with NSL3 binding in vivo (Fig. 3B). Interestingly, AT-rich sequences were highly enriched on promoters where TATA and other core promoter motifs were absent (Fig. 3B). We calculated the partial correlation coefficient of all 1024 5mers and found that AT content, rather than a specific motif, correlates best with NSL3 binding. Next, we used our MAPCap TSS as a reference to test whether the AT content is able to predict NSL targeting. To this end, we used the AT content of 61 29-bp windows around the MAPCap-based TSSs as predictors for NSL3 in vivo binding. We partitioned the genome into training sets and test sets, trained our logistic regression model with the genes in the training set, and applied the model to predict NSL3 binding on genes in the test sets (10-fold cross-validation). In this setting, the model correctly predicted 79% of true NSL3 targets and 76% of the non-NSL targets. The high AT content predicts the in vivo binding site of NSL3 for bins upstream of MAPCap TSSs. For bins overlapped with the +1 nucleosome position, high AT content correlates with lack of NSL3 binding (Fig. 3C). Furthermore, our analysis revealed that AT-rich sequence is a better predictor than DRE or other 5mer motifs (Fig. 3D,E; for a ROC curve, see Supplemental Fig. S5D). This result suggests that NSL3 recognizes AT-rich sequences in the genome. However, a comparison of the in vitro and in vivo patterns of NSL3 binding at MAPCap TSSs showed that in vitro binding of NSL3 is correlated with the AT content, whereas in vivo NSL3 binds downstream from the AT content peak (Supplemental Fig. S5E), suggesting that in vivo NSL3 binding is further refined by local chromatin context and other NSL complex members. Collectively, these results suggest that the AT content contributes to the targeting of the NSL complex to housekeeping promoters that lack canonical motifs such as TATA box or Inr.

### The NSL complex is required for TSS selection

**Figure 4.**
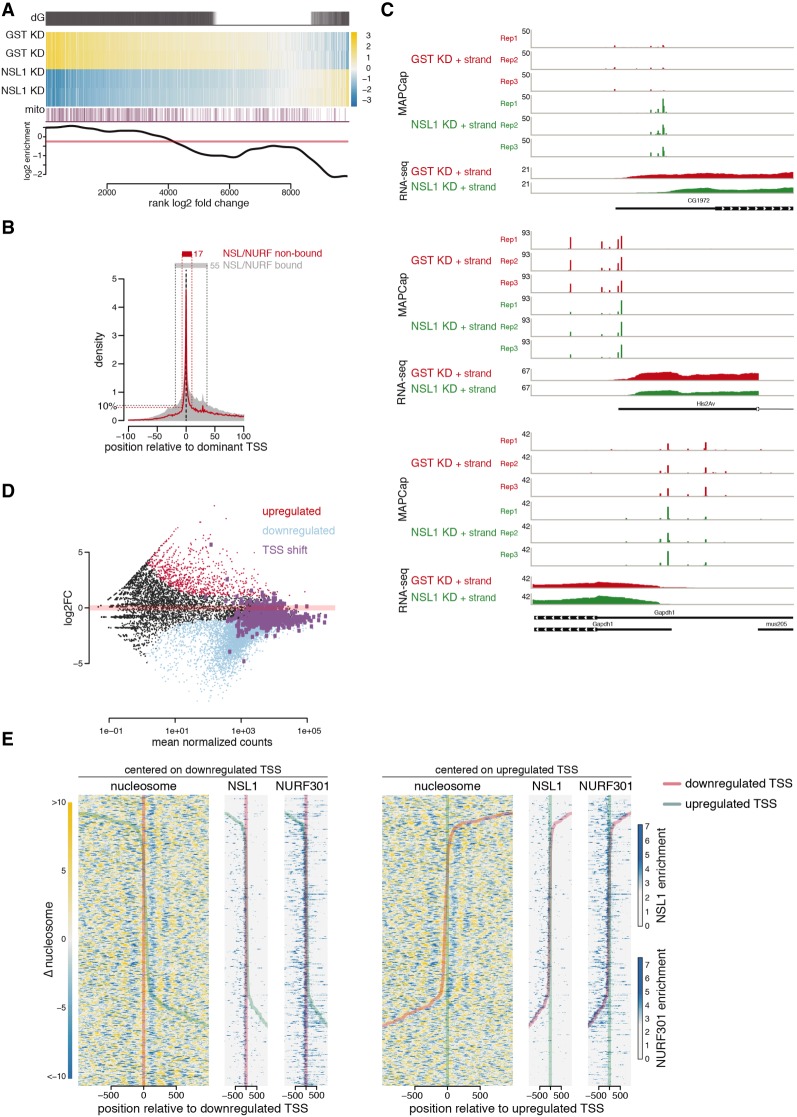
The NSL complex is required for TSS selection. (*A*) (dG) Differentially expressed genes as defined by DESeq2. (Heat map) Gene expression changes in control (GST) and NSL1-depleted cells; yellow and blue indicate up-regulation and down-regulation, respectively, compared with the average of all samples. (mito) Mitochondrial genes are marked as purple lines. (*Bottom*) The log_2_ enrichment of mitochondrial genes over the transcriptome as background. (*B*) The TSS signal obtained from MAPCap is plotted for NSL-bound (gray area) and non-NSL-bound (red line) genes. Signals for each gene are centered around the dominant TSS. The 10 percentile of signal accumulates within 17 bp from the dominant TSS for the NSL-nonbound genes, whereas the 10 percentile is spread across 55 bp for the NSL-bound genes. (*C*) Representative examples showing the MAPCap and RNA-seq data for the control (GST; red tracks) and NSL1 knockdown (green tracks). (*D*) MA plot showing the changes in gene expression upon NSL1 depletion. Light blue denotes down-regulated genes, whereas red denotes up-regulated genes. Genes displaying shifts in TSS selection upon NSL1 knockdown are marked in purple. (*E*) Heat maps showing change in nucleosome occupancy for genes displaying TSS shift in NSL1 knockdown, with yellow indicating an increase and blue a decrease in nucleosome signal. For each gene with a TSS shift event, one TSS of the gene is defined as down-regulated (red line in both heat maps), whereas another TSS of the same gene is labeled as up-regulated (green line) in the same gene. The *left* heat maps are centered at the down-regulated TSS (red). Heat maps on the *right* are centered at the up-regulated TSS (green). NSL1 and NURF301 ChIP-seq signals are shown for these 418 genes.

Consistent with our data, knockdown of NSL1 should have a strong effect on gene expression, and this is indeed what we observed in RNA sequencing (RNA-seq) experiments. The analysis revealed that 5225 (53%) of the 9850 genes for which we could detect expression in either control or NSL1 knockdown were significantly down-regulated at a false-discovery rate (FDR) of 10%, whereas only 774 (8%) were significantly up-regulated and 3851 (39%) were not significantly different (Supplemental Table S2; Supplemental Fig. S6C,D). When considering the log_2_ fold change, 7643 genes were down-regulated (Fig. 4A), indicating that the NSL complex is required for transcription of the vast majority of active genes. Notably, there was an overrepresentation of nuclear and mtDNA encoded mitochondrial proteins among the genes that were most down-regulated (Fig. 4A). The mammalian NSL complex has been reported to be required for the expression of respiratory genes from both nuclear and mtDNA (Chatterjee et al. 2016). To further investigate the effects on transcription specific to the promoter regions where nucleosome shift occurs, we used MAPCap analysis in wild-type and NSL1-depleted cells (Supplemental Table S3; Supplemental Fig. S6B): 4020 (64%) of the 6281 dominant MAPCap TSSs were significantly down-regulated at an FDR of 10%, whereas only 113 (2%) were significantly up-regulated and 2148 (34%) were not significantly changed. Clustering analysis revealed that the TSS expression and nucleosome changes correlate with NSL1 and NURF301 binding (Supplemental Fig. S6C), indicating that changes in the nucleosomal organization are correlated with the down-regulation of TSSs in a NSL1- and NURF301-dependent manner. Promoters targeted by NSL1 and NURF301 show a broader TSS pattern than do nonbound ones (Fig. 4B), which correlates with the absence of core promoter motifs (Schor et al. 2017) that are predominantly found in nonbound TSSs (Supplemental Fig. S6C).

If the TSS(s) are selected by the position of the +1 nucleosome, a delocalized +1 nucleosome may influence TSS firing and selection. We noticed cases in which TSS preference changes upon NSL1 knockdown (Fig. 4C). To identify genes that change their TSS preference upon NSL1 knockdown, we devised a statistical analysis (see the Materials and Methods) that identified 418 genes with a significant change in TSS usage at an FDR of 5%. Some of these genes alter their promoter usage, and 251 (60%) show changes in the TSS usage within a window of 200 bp, often with one TSS being favored whereas another is repressed. We asked how this change in TSS usage alters overall gene expression and found that six (1%) genes were up-regulated, 249 (60%) genes remained unchanged, and 162 (39%) genes were down-regulated (Fig. 4D). Overall expression of most of these genes remained unchanged, indicating that an alternate promoter or TSS compensates for the depressed NSL1-regulated TSSs. These genes were bound by both NSL1 and NURF301 (Fig. 4E). We therefore asked whether this change in TSS preference could be a direct consequence of the changes in the nucleosomal pattern upon NSL1 knockdown. For each of the 418 genes, we identified the TSS that is favored in NSL1 knockdown as an up-regulated TSS, whereas the TSS that showed reduced usage was marked as a down-regulated TSS. The down-regulated TSSs aligned with a local increase in nucleosome occupancy in their NDRs (Fig. 4E), indicating that the disruption of the canonical nucleosomal organization leads to a down-regulation of certain TSSs.

**Figure 5.**
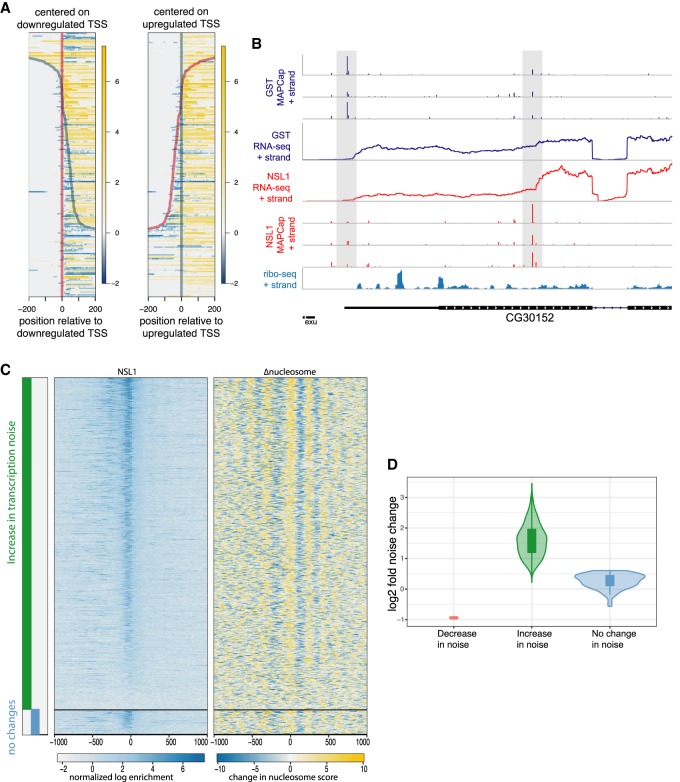
Transcription noise increases in the absence of the NSL complex. (*A*) Differential signal in RNA-seq comparing control and NSL1 knockdown at 5′ UTR regions. Heat maps are centered at the down-regulated (*left*) TSS and up-regulated TSS (*right*) as in Figure 4C. Yellow denotes an increase in RNA-seq read counts, whereas blue denotes a decrease in RNA-seq read counts and thus represent changes in the length of 5′ UTR. (*B*) Representative example showing MAPCap as well as RNA-seq data for control and NSL1 knockdown samples. Ribo-seq data from wild-type cells are provided (*bottom*). Ribo-seq data show upstream ORFs in the 5′ UTR region, where expression is reduced in NSL1 knockdown because of a shift in TSS selection. (*C*) (Leftmost) Bars showing genes with increased (green) and unchanged (blue) transcription variability. Differential variability was calculated using the BASiCS_TestDE function. The BASiCS_TestDE function also corrects for changes in gene expression between control and NSL1 knockdown. (*middle* and *right*). Heat maps showing NSL1 binding and changes in nucleosome occupancies upon NSL1 knockdown on the same genes. (*D*) Violin plot showing the change in transcription variability depicted by the green and blue bars in *C*.

The usage of an alternate promoter could have important consequences for the resulting mRNA. For example, a shift in TSS usage within a promoter can result in changes in 5′ untranslated region (UTR) length that may affect posttranscriptional regulation of the resulting mRNA (Hinnebusch et al. 2016; Leppek et al. 2018). We compared the RNA-seq coverage in these regions from control and NSL1 knockdown samples, focusing our analysis on genes that had RNA-seq coverage within a window of ±200 bp around the TSS. In cases when a downstream TSS was up-regulated, we clearly observed a reduction of RNA-seq coverage at the beginning of the 5′ UTR, indicating shorter transcripts. Likewise, when an upstream TSS was up-regulated, we observed more RNA-seq reads upstream of the 5′ UTR and hence a longer transcript (Fig. 5A). Consistently, the comparison of NSL1-mediated changes in TSS usage with Ribo-seq data (Dunn et al. 2013) revealed that TSS shifts upon loss of NSL1 would impact the 5′ UTR as well as the translated products in cases in which a TSS shift occurred downstream (Fig. 5B; Supplemental Fig. S6E). Thus, the NSL complex can influence TSS choice through canonical nucleosomal organization.

### Transcription noise increases in the absence of the NSL complex

Our results suggest that the NSL complex creates a transcription-competent nucleosome organization at its target promoters, enabling constitutive expression with concomitant low transcription noise (Eldar and Elowitz 2010; Lehner 2010; Munsky et al. 2012; Sanchez et al. 2013; Sharon et al. 2014; Ravarani et al. 2016). Disruption of this transcription-competent nucleosome organization at the TSS should lead to a TSS that requires chromatin remodeling before transcription such that it cycles between an off and an on state, which increases transcription noise (Eldar and Elowitz 2010; Munsky et al. 2012). Therefore, we asked whether the disruption of the nucleosome organization by the depletion of NSL1 results in an increase in transcription noise. For this purpose, we performed single-cell RNA-seq on control *Drosophila* S2 cells and cells depleted of NSL1 using the 10× genomics emulsion-based sequencing technology; 1753 cells and 4046 cells were sequenced for NSL-depleted and control samples, respectively. We selected cells with more than 2000 expressed genes and focused on a subset of genes that were expressed in >50% of the remaining cells. To detect changes in transcription noise. we used the BASiCS approach (Vallejos et al. 2015). Because transcription noise is influenced by transcription levels, we focused on 1008 genes that were not differentially expressed upon NSL1 knockdown and identified changes in transcription noise at an expected FDR of 10%. We observed increased transcription noise in most of these genes (Fig. 5C,D; Supplemental Fig. S7). Furthermore, the majority of genes with increased transcription noise were NSL1 targets and displayed disrupted nucleosome patterning upon NSL1 knockdown (Fig. 5C). Thus, our data suggest that the NSL complex is involved in suppressing transcriptional noise at target gene loci.

## Discussion

**Figure 6.**
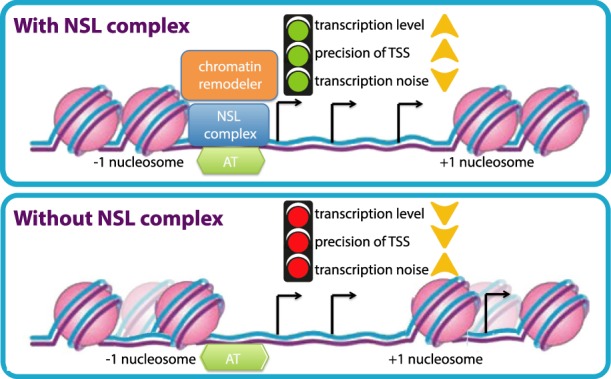
A schematic model. The NSL complex is required for nucleosome positioning at dispersed promoters of housekeeping genes. Genetic and biochemical analysis revealed that the NSL complex recruits NURF chromatin remodeling complex at target promoters. The NSL complex targets AT-rich sequences in TATA-less promoters. Loss of NSL complex not only leads to transcription down-regulation of targets but also affects the choice of TSSs of highly expressed genes with multiple TSSs. Loss of NSL complex also increases transcription noise at target genes. Thus, NSL complex plays a crucial role in transcription fidelity in the *Drosophila* genome.

In the present study, we set out to understand how transcription initiation is regulated on NSL-bound promoters, which are typically TATA-less housekeeping promoters with a dispersed TSS pattern. We uncover here that the NSL complex is required for maintaining the positioning of the +1 nucleosome at NSL-bound gene promoters, which is pivotal for not only effective transcription but also TSS fidelity in *Drosophila* (Fig. 6).

We find here that the NSL complex recruits the NURF chromatin remodeling complex to NSL target promoters. In mammals, BPTF (NURF301 homolog) binds to promoter-associated H3K4me2/3 and H4K16ac via its PHD and bromodomain, respectively, and the interaction is important for the recruitment of the NURF complex (Ruthenburg et al. 2007, 2011). In *Drosophila*, two isoforms of NURF301 exist: a longer isoform that encodes the C-terminal PHD and bromodomain, as well as a shorter isoform that lacks the two domains. The shorter isoform thus is unable to bind H3K4me3 or H4K16ac but is nevertheless sufficient to target the NURF complex to the majority of genes (Kwon et al. 2009), which then acts upon the +1 nucleosomes to properly position them. Our data suggest a plausible explanation on how the NURF complex may be recruited by transcription factors, such as the NSL complex, in addition to the histone marks. It is thus conceivable that the NURF complex interacts with both the NSL complex and the histone marks for accurate targeting to gene promoters.

Transcription stochasticity is known to play an instrumental role in processes such as cell differentiation, as well as cellular homeostasis in response to external stimuli (Eldar and Elowitz 2010; Munsky et al. 2012). TATA-containing promoters are believed to be noisier and more important for fate determination, whereas TATA-less promoters are considered to be less noisy and involved in cellular homeostasis (Shalek et al. 2013). However, it was previously not clear how TATA-less housekeeping genes regulate transcription noise levels. Our work here reveals that the NSL complex plays a role in the suppression of transcription noise at housekeeping genes. We show that nucleosome occupancies at NDR increase in the absence of the NSL complex. This increase likely posits that nucleosome occupancy of a certain promoter at a given time is more heterogenous between cells. Consistent with previous reports that showed that DNA sequences with low nucleosomal affinity display lower transcriptional noise (Dadiani et al. 2013; Sharon et al. 2014), our data suggest that the increase in heterogeneity of nucleosome occupancy leads to an increase in transcriptional noise as observed upon NSL depletion. Moreover, the NSL complex has been shown to facilitate the recruitment of Pol II machinery (Lam et al. 2012). Thus, it is also plausible that the NSL complex changes the dynamics of Pol II binding and initiation at chromatin and thereby represses transcriptional noise at NSL-bound genes. Our results have implication for further understanding how transcription noise can be regulated by transcription factors on distinct types of promoters.

Variants in core promoter DNA sequence affect TSS firing pattern in different *Drosophila* lines (Schor et al. 2017), yet it is not understood how these changes in DNA sequence are translated to changes in TSS pattern. Our MAPCap analyses in NSL1 knockdown cells reveal important insights into the crucial roles that the NSL complex binding and nucleosome positioning play in ensuring efficient TSS firing and selection. For most genes, TSS firing is compromised as nucleosomes invade the NDR in the absence of the NSL complex. Some genes compensate for the reduced TSS firing by up-regulating TSS firing from an alternative promoter of the same gene. This surprising result suggests that neighboring TSSs within one gene could rely on different mechanisms for initiation. It is thus possible that different TSSs may be preferred in different tissues or stress conditions in wild-type cells. This TSS preference shift may potentially be regulated by modulating nucleosome positioning or binding of the NSL complex. The misregulation in TSS preferences upon NSL1 knockdown is not without consequence. The TSS shift produces an RNA with altered 5′ UTR length and, in some cases, an altered 5′ nucleotide. The altered UTR length leads to different numbers of upstream open reading frames (uORFs) and RNA modifications in the UTR, whereas a changed starting nucleotide affects the frequency of m6Am modification. uORFs and RNA modifications have well-known effects on RNA stability and translation (Barbosa et al. 2013; Mauer et al. 2017), suggesting significant changes in the cellular proteome in the absence of the NSL complex.

Housekeeping TATA-less promoters are enriched in DNA motifs such as motifs 1, 6, and 7 and DRE (Ohler et al. 2002). Nevertheless, no single motif is enriched on the majority of housekeeping promoters, raising the following questions: Is there an equivalent to TBP/TATA box on housekeeping promoters, and how these promoters are targeted specifically? Our data suggest that the AT preference of NSL3 contributes to the targeting of the NSL complex to these TATA-less promoters. In line with this idea, we found that by using the AT content we can correctly predict 79% of true NSL3 in vivo targets and 76% of the non-NSL targets. However, these results also suggest that other targeting mechanisms have to act in parallel to achieve the in vivo binding pattern of the NSL complex. These targeting mechanisms could include DNA binding of other NSL complex members and interactions with other chromatin components such as histones and their modifications. Because the NSL complex binds to the majority of housekeeping promoters, our result suggests that the recognition of AT-rich sequences by NSL3 contributes to the discrimination of TATA-less promoters from the rest of the genome.

In summary, we show that the NSL complex functions to maintain a prominent promoter nucleosome pattern and subsequently guides TSS selection and suppresses transcription noise on dispersed housekeeping promoters. Our data also reveal that the NSL complex is recruited to the majority of TSSs that lack canonical promoter motifs such as the TATA-box and Inr by binding to AT-rich sequences. Taken together, this study provides a plausible explanation to the long-standing questions of how TATA-less promoters are recognized and transcribed. Based on our data, we propose a model whereby the NSL complex acts like a Swiss-army knife/platform to bring together the characteristic chromatin-modifying factors that are typically observed at housekeeping genes and required for their proper transcription.

## Materials and methods

### *Drosophila* rearing conditions and genetics

Unless otherwise specified, flies were reared on a standard cornmeal fly medium at 25°C, 70% relative humidity, and 12-h dark/12-h light cycle. For details regarding the genotypes, stocks, and genetic crossing schemes used in this study, please refer to the Supplemental Material.

### Knockdown experiments in S2 cells

The double-stranded RNAs against the NSL complex subunits and chromatin remodelers were designed to complement the exon sequences of the respective proteins with a length of 250–350 bp. These double-stranded RNAs did not show complementarity to other genes (with 18-bp seeds) besides the genes of interest, which were determined by E-RNAi (Horn and Boutros 2010). Double-stranded RNAs against the GST were used as a control. Further details regarding synthesis of dsRNAs are provided in the Supplemental Material.

For knockdown experiments, 2 mL of S2 cells at 1 million cells per milliliter was plated in six-well dishes and left to attach for 1 h. Ten micrograms of purified double-stranded RNA was diluted with Schneider's medium. The RNA transfection was performed using Lipofectamine RNAiMAX reagent (Life Technologies), according to the manufacturer's instructions. The double-stranded RNAs were mixed with the transfection reagent and kept for 15 min at room temperature before the mixture was added to the cells. The cells were harvested after 4 d, and cell numbers were counted.

The primers used for generating dsRNA are indicated in Supplemental Table S4.

### Luciferase assays

Briefly, 100 µL of cells at 1 million cells per milliliter was plated on 96-well plates. Cells in each well were transfected with a plasmid mixture of (1) 200 ng of pG5luc, which contains the firefly luciferase gene whose expression is controlled by UAS sequences; (2) 2 ng of pRL-hsp70, which contains a constitutively expressed Renilla luciferase gene; and (3) 50 ng of pAc5.1 vector containing either Gal4DBD-tagged NSL3 protein or Gal4DBD alone. Transfections were performed with X-tremeGENE DNA transfection reagents (Roche). After 2 d of incubation, cells were lysed (dual-luciferase kit, Promega), and luminescence was measured by using a Mithras plate reader (Berthold). For further details, see the Supplemental Material.

### ChIP

Briefly, S2 cells were cross-linked using 1.8% formaldehyde in crosslinking solution. After quenching with 125 mM glycine, the cells were washed with Paro 1, Paro 2, and RIPA buffers. The cells were then resuspended in RIPA buffer and were sheared using a Branson sonicator and Covaris sonicator. After verifying appropriate shearing, the chromatin was precleared using Protein A/Protein G beads. Appropriate antibodies were added to the chromatin and incubated overnight. Immunoprecipitation was performed using either Protein A or Protein G. The beads were washed thoroughly and reverse-crosslinked overnight at 65°C and treated with Proteinase K and RNase A. The DNA was purified using Minelute columns (Qiagen). For a detailed protocol, please refer to the Supplemental Material.

The primers used for quantitative PCR are listed in Supplemental Table S5.

### MNase-seq and Mnase-ChIP

Briefly, the cells were crosslinked with 1% formaldehyde in crosslinking solution. The samples were quenched using 125 mM glycine. NP-40 was used for cell permeabilization. The chromatin was then digested with MNase for 10 min at 25°C. The reaction was stopped by the addition of EDTA, NaCl, and SDS, and the samples were placed on ice. For ChIP, the following steps were the same as those mentioned in the ChIP protocol. For a detailed protocol, please refer to the Supplemental Material.

### MAPCap

Protein G magnetic Dynabeads (Life Technologies) were prepared with IPP buffer (50 mM Tris-HCl at pH 7.4, 150 mM NaCl, 0.1% NP-40). We incubated 2.5 µg of anti-m7G antibody (SYSY 201 011) with the beads for at least 1 h in 4°C. The beads were finally washed twice with IPP buffer. RNA extractions were performed with a Direct-zol miniprep kit (Zymo Research). Abundant small RNAs (<200 nt) were removed using a RNA clean and concentrator kit (Zymo Research) and eluted in 100 µL TE buffer (10 mM Tris-HCl at pH 8.0, 1 mM EDTA). RNA fragmentation was performed using a Covaris E220 focused-ultrasonicator (duty cycle, 10%; intensity, 5; power, 175 W; cycles/burst, 200; time, 140 sec). After sonication, the capped RNA was captured with the antibody-coupled Protein G magnetic beads for 1–2 h in 4°C. Then, the beads were washed three times with IPP buffer. RNA 3′ ends were repaired using PNK. Custom-made barcoded adapters were ligated to the RNA using T4 RNA ligase 1 for 1 h at 25°C. Excess adapters were washed away with IPP buffer, and RNA was purified by column purification. Isolated RNA was reverse-transcribed and treated with RNase H. cDNA was column-purified and circularized with CircLigase for 2–16 h. Circularized cDNA was directly PCR amplified; the amplified library was finally cleaned up using AMPure beads. For further information regarding MAPCap analysis, please refer to the Supplemental Material.

### Computational analysis

For a thorough description of the ChIP-seq, MAPCap, MNase-seq, scRNA-seq, gDNA-IP-seq, and RNA-seq analysis, please refer to the Supplemental Material.

### Data accession

All of the genome-wide data sets in this manuscript have been deposited in Gene Expression Omnibus under accession number GSE118726

## Supplementary Material

Supplemental Material

## References

[GAD321489LAMC1] Alkhatib SG, Landry JW. 2011 The nucleosome remodeling factor. FEBS Lett 585: 3197–3207. 10.1016/j.febslet.2011.09.003 21920360 PMC4839296

[GAD321489LAMC2] Barbosa C, Peixeiro I, Romão L. 2013 Gene expression regulation by upstream open reading frames and human disease. PLoS Genet 9: e1003529 10.1371/journal.pgen.1003529 23950723 PMC3738444

[GAD321489LAMC3] Cai Y, Jin J, Gottschalk AJ, Yao T, Conaway JW, Conaway RC. 2006 Purification and assay of the human INO80 and SRCAP chromatin remodeling complexes. Methods 40: 312–317. 10.1016/j.ymeth.2006.06.023 17101442 PMC3092633

[GAD321489LAMC4] Cai Y, Jin J, Swanson SK, Cole MD, Choi SH, Florens L, Washburn MP, Conaway JW, Conaway RC. 2010 Subunit composition and substrate specificity of a MOF-containing histone acetyltransferase distinct from the male-specific lethal (MSL) complex. J Biol Chem 285: 4268–4272. 10.1074/jbc.C109.087981 20018852 PMC2836030

[GAD321489LAMC5] Carninci P, Sandelin A, Lenhard B, Katayama S, Shimokawa K, Ponjavic J, Semple CA, Taylor MS, Engström PG, Frith MC, 2006 Genome-wide analysis of mammalian promoter architecture and evolution. Nat Genet 38: 626–635. 10.1038/ng1789 16645617

[GAD321489LAMC6] Chatterjee A, Seyfferth J, Lucci J, Gilsbach R, Preissl S, Böttinger L, Mårtensson CU, Panhale A, Stehle T, Kretz O, 2016 MOF acetyl transferase regulates transcription and respiration in mitochondria. Cell 167: 722–738 e723. 10.1016/j.cell.2016.09.052 27768893

[GAD321489LAMC7] Chelmicki T, Dündar F, Turley MJ, Khanam T, Aktas T, Ramírez F, Gendrel AV, Wright PR, Videm P, Backofen R, 2014 MOF-associated complexes ensure stem cell identity and *Xist* repression. Elife 3: e02024 10.7554/eLife.02024 24842875 PMC4059889

[GAD321489LAMC8] Chen L, Cai Y, Jin J, Florens L, Swanson SK, Washburn MP, Conaway JW, Conaway RC. 2011 Subunit organization of the human INO80 chromatin remodeling complex: An evolutionarily conserved core complex catalyzes ATP-dependent nucleosome remodeling. J Biol Chem 286: 11283–11289. 10.1074/jbc.M111.222505 21303910 PMC3064184

[GAD321489LAMC9] Chereji RV, Kan TW, Grudniewska MK, Romashchenko AV, Berezikov E, Zhimulev IF, Guryev V, Morozov AV, Moshkin YM. 2016 Genome-wide profiling of nucleosome sensitivity and chromatin accessibility in *Drosophila melanogaster*. Nucleic Acids Res 44: 1036–1051. 10.1093/nar/gkv978 26429969 PMC4756854

[GAD321489LAMC10] Contrino S, Smith RN, Butano D, Carr A, Hu F, Lyne R, Rutherford K, Kalderimis A, Sullivan J, Carbon S, 2012 modMine: flexible access to modENCODE data. Nucleic Acids Res 40: D1082–D1088. 10.1093/nar/gkr921 22080565 PMC3245176

[GAD321489LAMC11] Corona DFV, Längst G, Clapier CR, Bonte EJ, Ferrari S, Tamkun JW, Becker PB. 1999 ISWI is an ATP-dependent nucleosome remodeling factor. Mol Cell 3: 239–245. 10.1016/S1097-2765(00)80314-7 10078206

[GAD321489LAMC12] Dadiani M, van Dijk D, Segal B, Field Y, Ben-Artzi G, Raveh-Sadka T, Levo M, Kaplow I, Weinberger A, Segal E. 2013 Two DNA-encoded strategies for increasing expression with opposing effects on promoter dynamics and transcriptional noise. Genome Res 23: 966–976. 10.1101/gr.149096.112 23403035 PMC3668364

[GAD321489LAMC13] Deuring R, Fanti L, Armstrong JA, Sarte M, Papoulas O, Prestel M, Daubresse G, Verardo M, Moseley SL, Berloco M, 2000 The ISWI chromatin-remodeling protein is required for gene expression and the maintenance of higher order chromatin structure in vivo. Mol Cell 5: 355–365. 10.1016/S1097-2765(00)80430-X 10882076

[GAD321489LAMC14] Dunn JG, Foo CK, Belletier NG, Gavis ER, Weissman JS. 2013 Ribosome profiling reveals pervasive and regulated stop codon readthrough in *Drosophila melanogaster*. Elife 2: e01179 10.7554/eLife.01179 24302569 PMC3840789

[GAD321489LAMC15] Dutta A, Workman JL. 2012 Nucleosome positioning: multiple mechanisms toward a unifying goal. Mol Cell 48: 1–2. 10.1016/j.molcel.2012.09.015 23062951

[GAD321489LAMC16] Eldar A, Elowitz MB. 2010 Functional roles for noise in genetic circuits. Nature 467: 167–173. 10.1038/nature09326 20829787 PMC4100692

[GAD321489LAMC17] Feller C, Prestel M, Hartmann H, Straub T, Söding J, Becker PB. 2012 The MOF-containing NSL complex associates globally with housekeeping genes, but activates only a defined subset. Nucleic Acids Res 40: 1509–1522. 10.1093/nar/gkr869 22039099 PMC3287193

[GAD321489LAMC18] Gilissen C, Hehir-Kwa JY, Thung DT, van de Vorst M, van Bon BW, Willemsen MH, Kwint M, Janssen IM, Hoischen A, Schenck A, 2014 Genome sequencing identifies major causes of severe intellectual disability. Nature 511: 344–347. 10.1038/nature13394 24896178

[GAD321489LAMC19] Haberle V, Li N, Hadzhiev Y, Plessy C, Previti C, Nepal C, Gehrig J, Dong X, Akalin A, Suzuki AM, 2014 Two independent transcription initiation codes overlap on vertebrate core promoters. Nature 507: 381–385. 10.1038/nature12974 24531765 PMC4820030

[GAD321489LAMC20] Hinnebusch AG, Ivanov IP, Sonenberg N. 2016 Translational control by 5′-untranslated regions of eukaryotic mRNAs. Science 352: 1413–1416. 10.1126/science.aad9868 27313038 PMC7422601

[GAD321489LAMC21] Horn T, Boutros M. 2010 E-RNAi: a web application for the multi-species design of RNAi reagents—2010 update. Nucleic Acids Res 38: W332–W339. 10.1093/nar/gkq317 20444868 PMC2896145

[GAD321489LAMC22] Kadonaga JT. 2012 Perspectives on the RNA polymerase II core promoter. Wiley Interdiscip Rev Dev Biol 1: 40–51. 10.1002/wdev.21 23801666 PMC3695423

[GAD321489LAMC23] Koolen DA, Kramer JM, Neveling K, Nillesen WM, Moore-Barton HL, Elmslie FV, Toutain A, Amiel J, Malan V, Tsai AC, 2012 Mutations in the chromatin modifier gene KANSL1 cause the 17q21.31 microdeletion syndrome. Nat Genet 44: 639–641. 10.1038/ng.2262 22544363

[GAD321489LAMC24] Kwon SY, Xiao H, Wu C, Badenhorst P. 2009 Alternative splicing of NURF301 generates distinct NURF chromatin remodeling complexes with altered modified histone binding specificities. PLoS Genet 5: e1000574 10.1371/journal.pgen.1000574 19629165 PMC2705796

[GAD321489LAMC25] Kwon SY, Grisan V, Jang B, Herbert J, Badenhorst P. 2016 Genome-wide mapping targets of the metazoan chromatin remodeling factor NURF reveals nucleosome remodeling at enhancers, core promoters and gene insulators. PLoS Genet 12: e1005969 10.1371/journal.pgen.1005969 27046080 PMC4821604

[GAD321489LAMC26] Lai WKM, Pugh BF. 2017 Understanding nucleosome dynamics and their links to gene expression and DNA replication. Nat Rev Mol Cell Biol 18: 548–562. 10.1038/nrm.2017.47 28537572 PMC5831138

[GAD321489LAMC27] Lam KC, Mühlpfordt F, Vaquerizas JM, Raja SJ, Holz H, Luscombe NM, Manke T, Akhtar A. 2012 The NSL complex regulates housekeeping genes in *Drosophila*. PLoS Genet 8: e1002736 10.1371/journal.pgen.1002736 22723752 PMC3375229

[GAD321489LAMC28] Lehner B. 2010 Conflict between noise and plasticity in yeast. PLoS Genet 6: e1001185 10.1371/journal.pgen.1001185 21079670 PMC2973811

[GAD321489LAMC29] Leppek K, Das R, Barna M. 2018 Functional 5′ UTR mRNA structures in eukaryotic translation regulation and how to find them. Nat Rev Mol Cell Biol 19: 158–174. 10.1038/nrm.2017.103 29165424 PMC5820134

[GAD321489LAMC30] Mauer J, Luo X, Blanjoie A, Jiao X, Grozhik AV, Patil DP, Linder B, Pickering BF, Vasseur JJ, Chen Q, 2017 Reversible methylation of m^6^A_m_ in the 5′ cap controls mRNA stability. Nature 541: 371–375. 10.1038/nature21022 28002401 PMC5513158

[GAD321489LAMC31] Mendjan S, Taipale M, Kind J, Holz H, Gebhardt P, Schelder M, Vermeulen M, Buscaino A, Duncan K, Mueller J, 2006 Nuclear pore components are involved in the transcriptional regulation of dosage compensation in *Drosophila*. Mol Cell 21: 811–823. 10.1016/j.molcel.2006.02.007 16543150

[GAD321489LAMC32] Munsky B, Neuert G, van Oudenaarden A. 2012 Using gene expression noise to understand gene regulation. Science 336: 183–187. 10.1126/science.1216379 22499939 PMC3358231

[GAD321489LAMC33] Ni T, Corcoran DL, Rach EA, Song S, Spana EP, Gao Y, Ohler U, Zhu J. 2010 A paired-end sequencing strategy to map the complex landscape of transcription initiation. Nat Methods 7: 521–527. 10.1038/nmeth.1464 20495556 PMC3197272

[GAD321489LAMC34] Ohler U, Liao GC, Niemann H, Rubin GM. 2002 Computational analysis of core promoters in the *Drosophila* genome. Genome Biol 3: RESEARCH0087 10.1186/gb-2002-3-12-research0087 12537576 PMC151189

[GAD321489LAMC35] Rach EA, Yuan HY, Majoros WH, Tomancak P, Ohler U. 2009 Motif composition, conservation and condition-specificity of single and alternative transcription start sites in the *Drosophila* genome. Genome Biol 10: R73 10.1186/gb-2009-10-7-r73 19589141 PMC2728527

[GAD321489LAMC36] Raja SJ, Charapitsa I, Conrad T, Vaquerizas JM, Gebhardt P, Holz H, Kadlec J, Fraterman S, Luscombe NM, Akhtar A. 2010 The nonspecific lethal complex is a transcriptional regulator in *Drosophila*. Mol Cell 38: 827–841. 10.1016/j.molcel.2010.05.021 20620954

[GAD321489LAMC37] Ravarani CN, Chalancon G, Breker M, de Groot NS, Babu MM. 2016 Affinity and competition for TBP are molecular determinants of gene expression noise. Nat Commun 7: 10417 10.1038/ncomms10417 26832815 PMC4740812

[GAD321489LAMC38] Ravens S, Fournier M, Ye T, Stierle M, Dembele D, Chavant V, Tora L. 2014 Mof-associated complexes have overlapping and unique roles in regulating pluripotency in embryonic stem cells and during differentiation. Elife 3: e02104 10.7554/eLife.02104 PMC4059888 24898753

[GAD321489LAMC39] Ruthenburg AJ, Li H, Patel DJ, Allis CD. 2007 Multivalent engagement of chromatin modifications by linked binding modules. Nat Rev Mol Cell Biol 8: 983–994. 10.1038/nrm2298 18037899 PMC4690530

[GAD321489LAMC40] Ruthenburg AJ, Li H, Milne TA, Dewell S, McGinty RK, Yuen M, Ueberheide B, Dou Y, Muir TW, Patel DJ, 2011 Recognition of a mononucleosomal histone modification pattern by BPTF via multivalent interactions. Cell 145: 692–706. 10.1016/j.cell.2011.03.053 21596426 PMC3135172

[GAD321489LAMC41] Sanchez A, Choubey S, Kondev J. 2013 Regulation of noise in gene expression. Annu Rev Biophys 42: 469–491. 10.1146/annurev-biophys-083012-130401 23527780

[GAD321489LAMC42] Schor IE, Degner JF, Harnett D, Cannavò E, Casale FP, Shim H, Garfield DA, Birney E, Stephens M, Stegle O, 2017 Promoter shape varies across populations and affects promoter evolution and expression noise. Nat Genet 49: 550–558. 10.1038/ng.3791 28191888

[GAD321489LAMC43] Shalek AK, Satija R, Adiconis X, Gertner RS, Gaublomme JT, Raychowdhury R, Schwartz S, Yosef N, Malboeuf C, Lu D, 2013 Single-cell transcriptomics reveals bimodality in expression and splicing in immune cells. Nature 498: 236–240. 10.1038/nature12172 23685454 PMC3683364

[GAD321489LAMC44] Sharon E, van Dijk D, Kalma Y, Keren L, Manor O, Yakhini Z, Segal E. 2014 Probing the effect of promoters on noise in gene expression using thousands of designed sequences. Genome Res 24: 1698–1706. 10.1101/gr.168773.113 25030889 PMC4199362

[GAD321489LAMC45] Spradling AC, Stern D, Beaton A, Rhem EJ, Laverty T, Mozden N, Misra S, Rubin GM. 1999 The Berkeley *Drosophila* Genome Project gene disruption project: single P-element insertions mutating 25% of vital *Drosophila* genes. Genetics 153: 135–177. 10471706 10.1093/genetics/153.1.135 PMC1460730

[GAD321489LAMC46] Struhl K, Segal E. 2013 Determinants of nucleosome positioning. Nat Struct Mol Biol 20: 267–273. 10.1038/nsmb.2506 23463311 PMC3740156

[GAD321489LAMC47] Vallejos CA, Marioni JC, Richardson S. 2015 BASiCS: Bayesian analysis of single-cell sequencing data. PLoS Comput Biol 11: e1004333 10.1371/journal.pcbi.1004333 26107944 PMC4480965

[GAD321489LAMC48] Vo Ngoc V, Wang YL, Kassavetis GA, Kadonaga JT. 2017 The punctilious RNA polymerase II core promoter. Genes Dev 31: 1289–1301. 10.1101/gad.303149.117 28808065 PMC5580651

[GAD321489LAMC49] Workman JL, Roeder RG. 1987 Binding of transcription factor TFIID to the major late promoter during in vitro nucleosome assembly potentiates subsequent initiation by RNA polymerase II. Cell 51: 613–622. 10.1016/0092-8674(87)90130-9 3677170

[GAD321489LAMC50] Yamamoto YY, Yoshitsugu T, Sakurai T, Seki M, Shinozaki K, Obokata J. 2009 Heterogeneity of *Arabidopsis* core promoters revealed by high-density TSS analysis. Plant J 60: 350–362. 10.1111/j.1365-313X.2009.03958.x 19563441

[GAD321489LAMC51] Yu L, Song Y, Wharton RP. 2010 E(nos)/CG4699 required for *nanos* function in the female germ line of *Drosophila*. Genesis 48: 161–170. 10.1002/dvg.20600 20095054

[GAD321489LAMC52] Zollino M, Orteschi D, Murdolo M, Lattante S, Battaglia D, Stefanini C, Mercuri E, Chiurazzi P, Neri G, Marangi G. 2012 Mutations in *KANSL1* cause the 17q21.31 microdeletion syndrome phenotype. Nat Genet 44: 636–638. 10.1038/ng.2257 22544367

