# Supplementary material for: The NSL complex-mediated nucleosome landscape is required to maintain transcription fidelity and suppression of transcription noise
Source: Genes Dev. 2019 Apr 1;33(7-8):452–65. doi: 10.1101/gad.321489.118 (PMC6446542; doi:10.1101/gad.321489.118)
Supplement: Supplemental Material [file supp_33_7-8_452__index.html]

The NSL complex-mediated nucleosome landscape is required to maintain transcription fidelity and suppression of transcription noise — Supplemental Material 

# The NSL complex-mediated nucleosome landscape is required to maintain transcription fidelity and suppression of transcription noise

## Supplemental Material

- Supplemental\_Information.docx
- Supplemental\_Table\_S3.xlsx
- Supplemental\_Table\_S1.xlsx
- Supplemental\_Table\_S2.xlsx
