# Supplementary material for: The NSL complex-mediated nucleosome landscape is required to maintain transcription fidelity and suppression of transcription noise
Source: Genes Dev. 2019 Apr 1;33(7-8):452–65. doi: 10.1101/gad.321489.118 (PMC6446542; doi:10.1101/gad.321489.118)
Supplement: Supplemental Material [file supp_gad.321489.118_Supplemental_Information.docx]

**The NSL complex mediated nucleosome landscape is required to maintain transcription fidelity and suppression of transcription noise**

Kin Chung Lam^1,ϕ^, Ho-Ryun Chung^2,3ϕ^, Giuseppe Semplicio^1^, Shantanu S. Iyer^1^_,_ Aline Gaub^1,5^, Vivek Bhardwaj^1^, Herbert Holz^1^, Plamen Georgiev^1^ and Asifa Akhtar^1,^*

**Supplemental Information**

This file contains:

7 Supplemental figures and figure legends

2 Supplemental tables

Materials and Methods


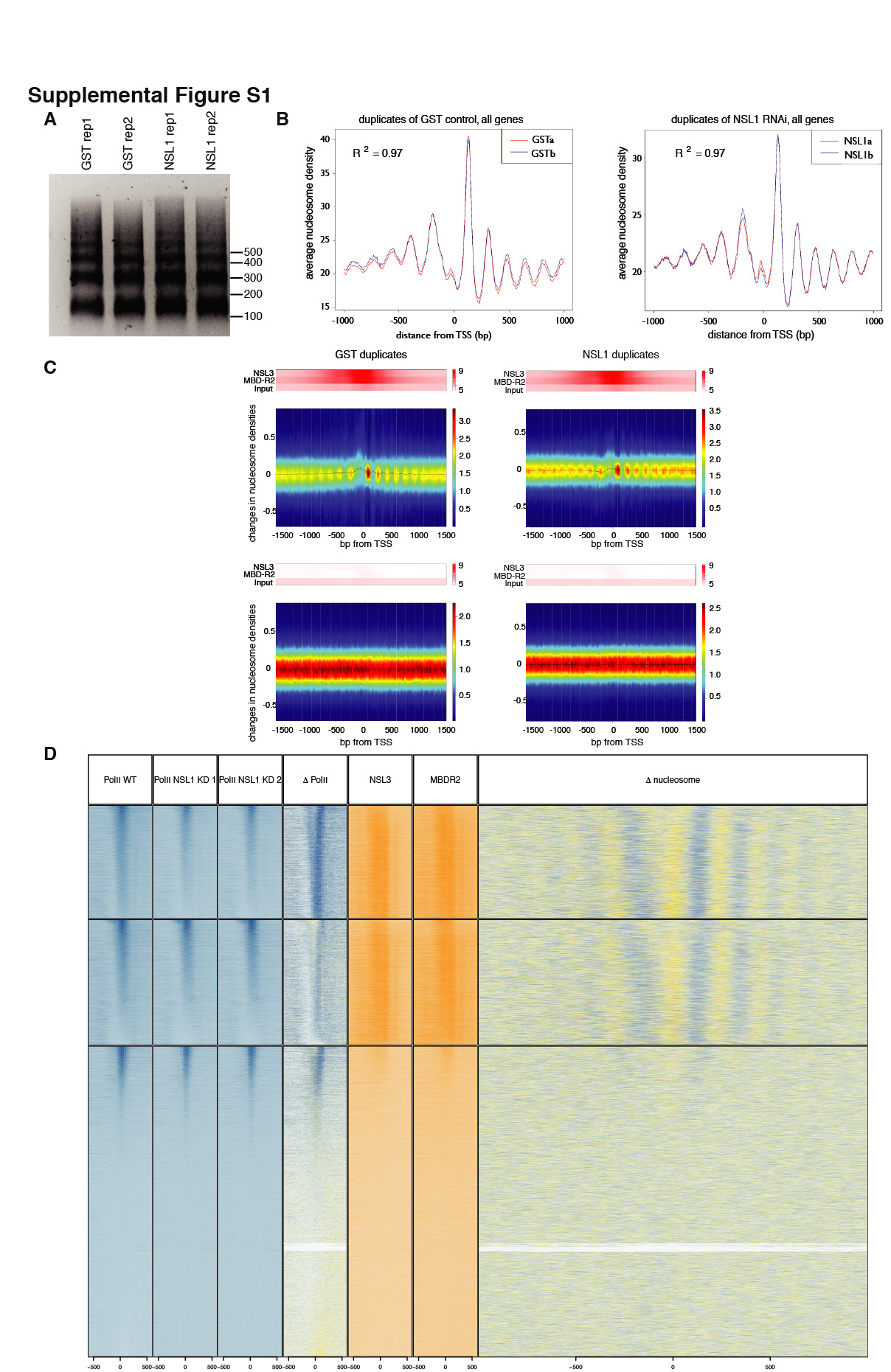


**Supplemental Figure S1**

(A) Digestion pattern of samples prepared for MNase-seq experiments. Knockdown experiments using dsRNA against GST (control) NSL1 are done in duplicates.

(B) Summary plots showing correlation between duplicates in MNase-seq experiments. R^2^ values are calculated from Spearman correlation coefficient. A region 1 kb around the TSS is shown.

(C) Heatmap showing the differences in nucleosome densities between replicates of control (GST; left) and NSL1 KD (right) for active (above) and inactive (below) genes. The y-axis represents changes in nucleosome signals. The color scale bar indicates scatter density. ChIP-seq signals of NSL3 and MBD-R2 are represented in bars above the heatmap to show the binding sites of the proteins. A region 1.5 kb around the TSS is shown.

(D) Heatmaps from left to right, log2 Pol II enrichment over input for WT, NSL1 KD replicate 1, NSL1 KD replicate 2, the difference between the average Pol II signal of NSL1 KD replicates 1 and 2 and the WT, log2 NSL3 enrichment over input, log2 MBDR2 enrichment over input and the change in nucleosome signal. The x-axis indicates the position with respect to the TSS in base pairs.


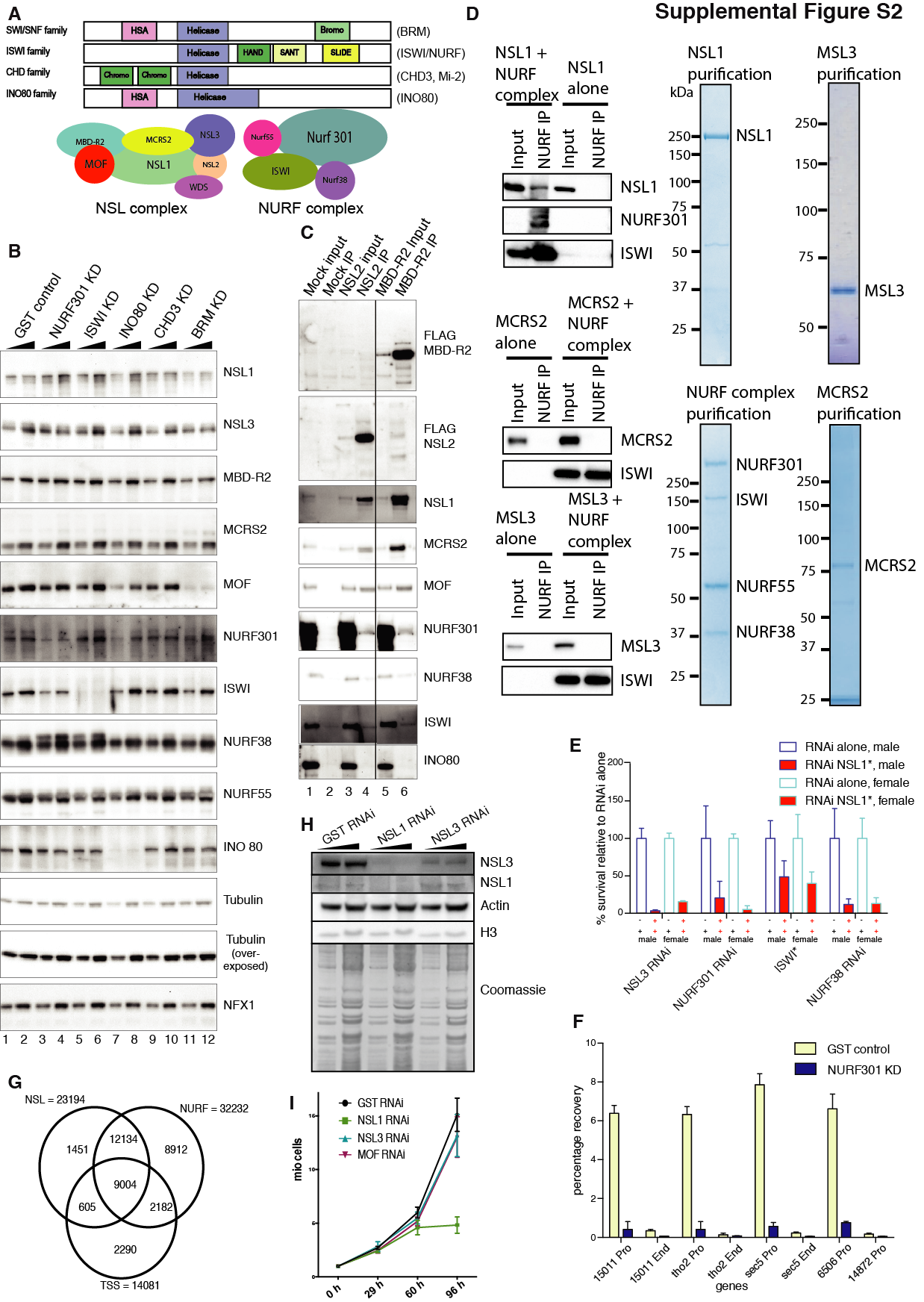


**Supplemental Figure S2**

(A) (Top) Schematic representation of 4 families of chromatin remodelers. Proteins of the respective family used in this study are shown on the right. (Bottom) Cartoon representation of core NSL complex and NURF complex members.

(B) Western blot showing protein levels of the NSL complex and chromatin remodelers in NURF301, ISWI, INO80 and CHD3 KD. The western blots show that the KD efficiently deplete the respective remodelers. The level of MOF is significantly reduced in the BRM KD.

(C) Western blot analysis of immunoprecipitation of exogenous FLAG-tagged NSL2 and MBD-R2 proteins using anti-FLAG peptides antibody. The protein bands are detected with antibodies against the NSL complex members, NURF38, NURF301, ISWI and INO80.

(D) Western blot analysis of immunoprecipitation of *in vitro* expressed HA-tagged ISWI using anti-HA peptides antibody. The NURF complex, including HA-ISWI, was incubated with NSL1, MCRS2 (member shared by the INO80 and NSL complex) or MSL3 (member of the MSL/MOF complex). Coomassie protein gels show individual purification of NSL1, MSL3, MCRS2 and NURF complex used in the experiment. KDa denotes molecular weight markers.

(E) Genetic interaction between the NSL and the NURF complex as in Figure 2D. NSL1 mutant shows genetic interaction with NURF301, ISWI and NURF38 in both males and females.

(F) ChIP-qPCR analysis using NURF301 antibody in wild type and NURF301-depleted S2 cells. The antibody specifically detects NURF301 signal in the wild type but not in the KD samples. Percentage of input recovery is shown on the y-axis.

(G) Venn diagram showing overlapping binding sites of NSL1 and NURF301 as detected by ChIP-seq experiments.

(H) Western blot showing the protein levels of NSL1 and NSL3, along with loading controls, upon either NSL1 or NSL3 RNAi. The Western blot shows that the knockdown sufficiently depleted the respective components of the NSL complex.

(I) A growth curve analysis at 0, 29, 60 and 96h after knockdown. It shows that NSL3 and MOF RNAi affects the growth of the cells only mildly. NSL1 knockdown causes the cells to grow slower after the 60h time point.


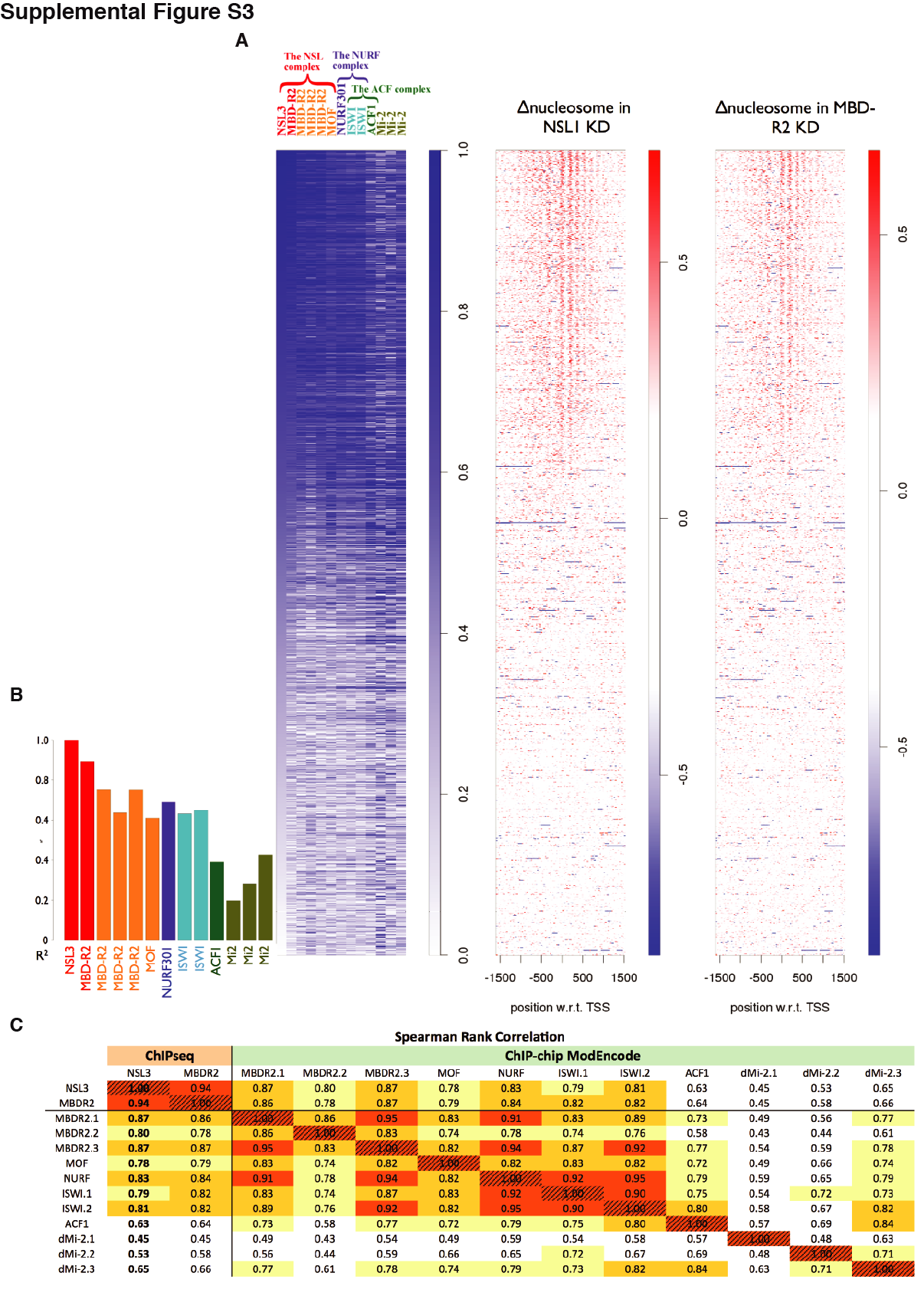


**Supplemental Figure S3**

(A) The NSL complex binding sites are more similar to those of the NURF complex than those of ACF1 or Mi-2 (leftmost heatmap) Heatmap showing the promoter (TSS±200 bp) binding signal of (left to right) NSL3 and MBD-R2 (ChIP-seq, (Lam et al. 2012)) as well as MBD-R2 (in triplicates), MOF, NURF301, ISWI (in duplicates), ACF1 and Mi-2 (in triplicates) (ModENCODE). The intensity of the blue color indicates the rank of enrichment signal. (central and rightmost) Heatmaps showing the changes in nucleosome density on the respective genes for the region TSS ± 1.5 kb. The intensity of the red color indicates the degree of nucleosome loss. All heatmaps were in the same gene order arranged according to the NSL3 (ChIP-seq) enrichment.

(B) The bar chart displays the spearman correlation coefficients of the NSL3 ranks against ranks in other tracks. There are high correlations between binding of NSL3 and the binding of NURF301 and ISWI and NSL3 binding is less correlated with the ACF1 (also interact with ISWI) or Mi-2.

(C) All pairwise Spearman rank correlation coefficients can be found in the table. Data in the first column of the table, where the numbers are in bold, was taken to plot the bar chart mentioned above. The table is color coded: with red (0.9) and orange (0.8) represent highly correlated pairs of profiles, while yellow (0.7) and white (<0.7) represent lowly correlated pairs. All tracks are ChIP-chip data from ModENCODE, unless otherwise stated. These data are publicly available and were retrieved from the ModENCODE project website (www.modencode.org)


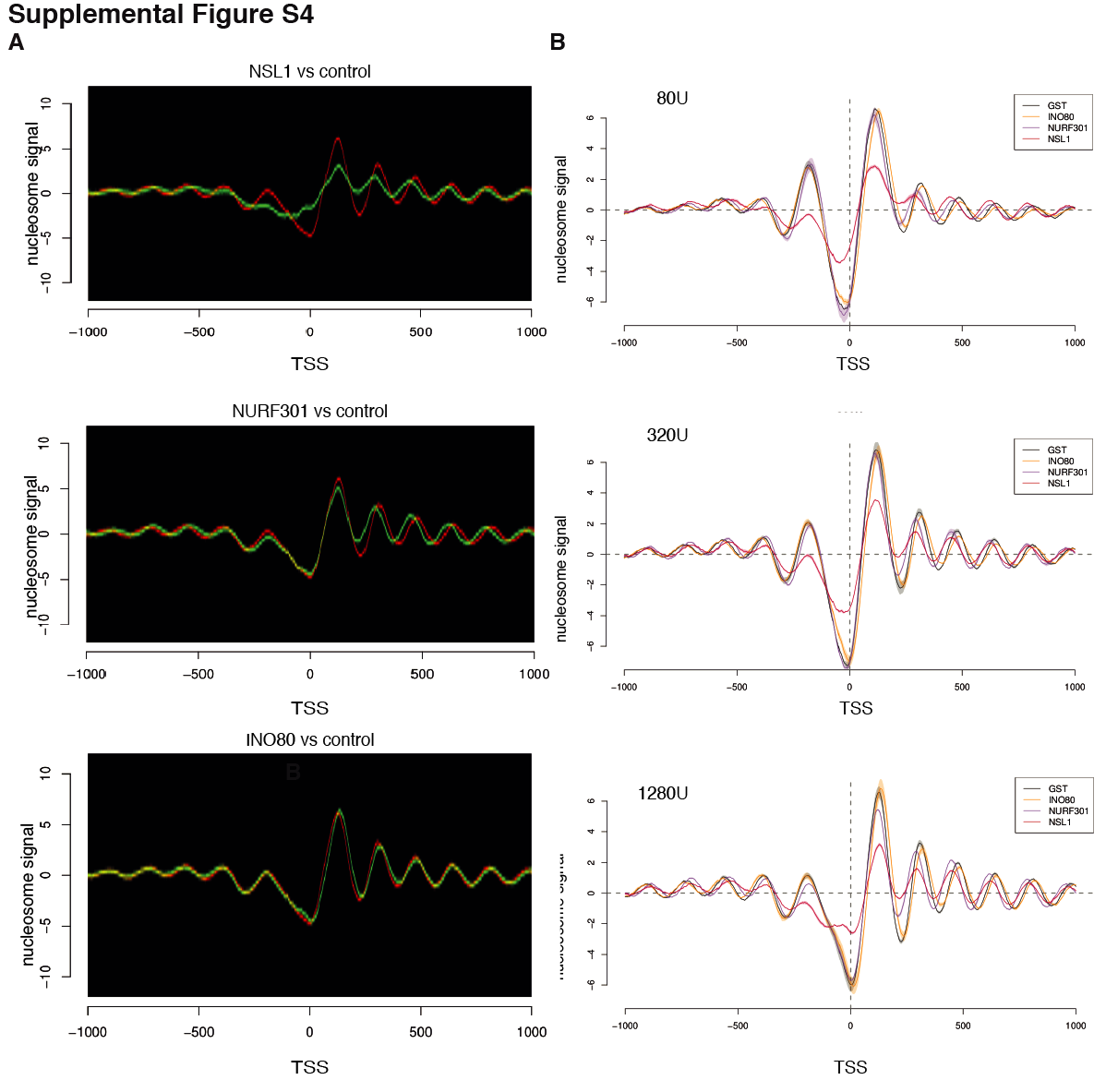


**Supplemental Figure S4**

(A) Summary plot showing nucleosome shift in NSL1, INO80 and NURF301 KD. Nucleosome signals of MNase-seq are plotted on the y-axis. Red lines show the control experiments and green lines show the KD experiments. A region 1 kb around the TSS is shown.

(B) MNase-seq experiments were reproduced with different degree of MNase digestions. Mnase-seq experiments are repeated with 80, 320 and 1280U of MNase in control, NSL1, NURF301 and INO80 depleted cells. The shaded area around the lines represent the variation between replicates.


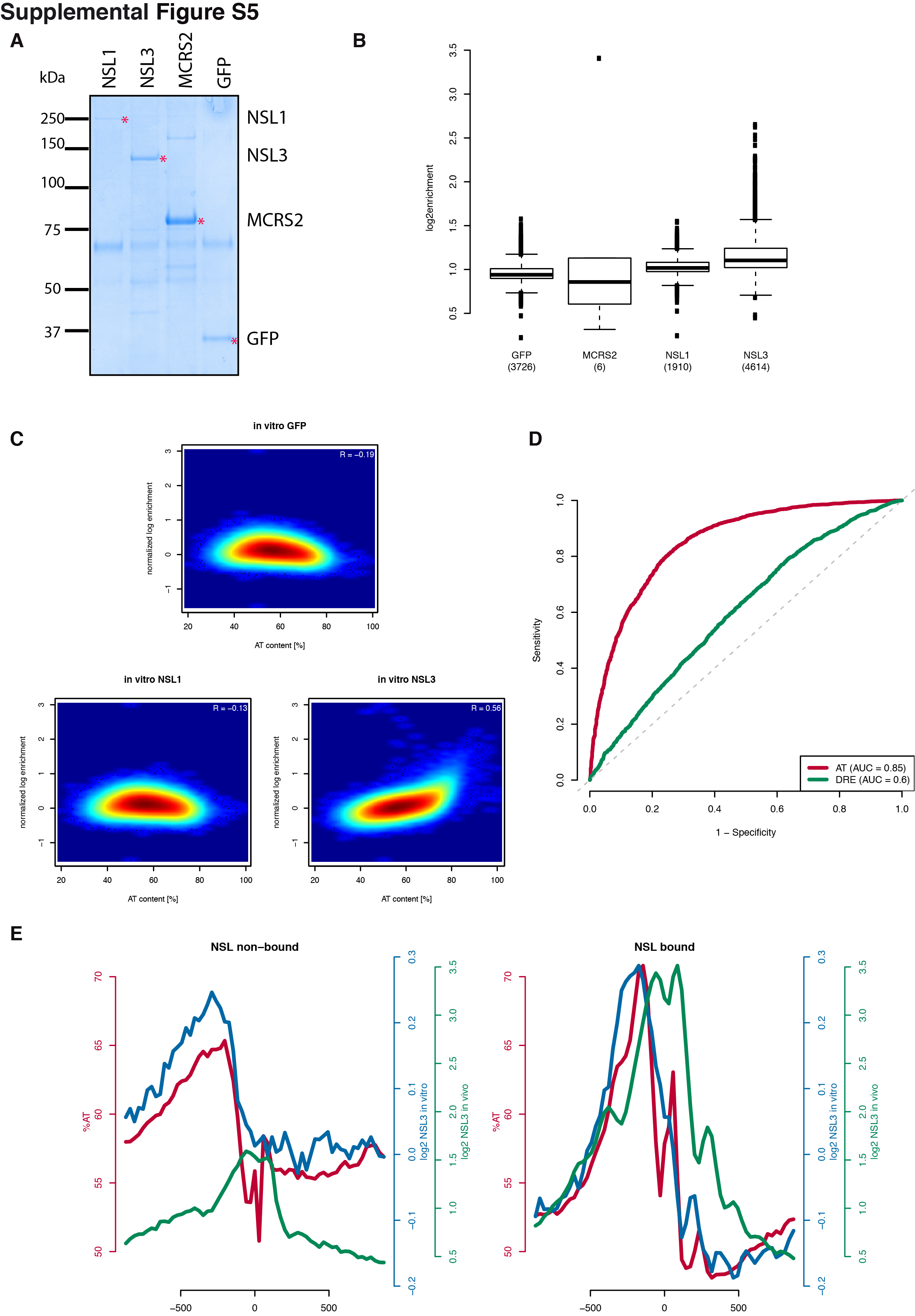


**Supplemental Figure S5**

(A) Protein gels showing purification of NSL1, NSL3, MCRS2 and GFP used for the genomic DNA immunoprecipitation experiment. KDa represents molecular weight markers.

(B) gDNA peaks for GFP, MCRS2, NSL1 and NSL3. On the x-axis the baits for the gDNA pulldowns are shown; number in parentheses indicate the number of peaks. On the y-axis the normalized log2 enrichment over input is shown.

(C) Scatter plots showing correlation between *in vitro* binding of GFP, NSL1, NSL3 and AT content.

(D) ROC curves for the prediction using the AT-content (red) in 61 29-base pair bins around the MAPCap TSSs, and the DRE motif score (green) in a window  -70 to -10 upstream of the MAPCap TSSs. Shown on x-axis is 1 - Specificity, i.e. the false positive rate. Shown on the y-axis is sensitivity, i.e. true positive rate. The dashed diagonal line indicates complete random prediction. AUC denotes the area under the curve, which had been 0.85 for the AT-content and 0.6 for the DRE motif score.

(E) Shown are the average profiles of the AT content in percent, log2 fold enrichment of NSL3 in vitro (blue) and log2 fold enrichment of NSL3 in vivo (green), for NSL non-bound (left) and NSL-bound (right) MAPCap TSSs. The x-axis indicates the position with respect to the TSS in base pairs, the y-axis denote the AT-content (red), the log2 NSL3 enrichment *in vitro* (blue) and the log2 NSL3 enrichment *in vivo* (green).


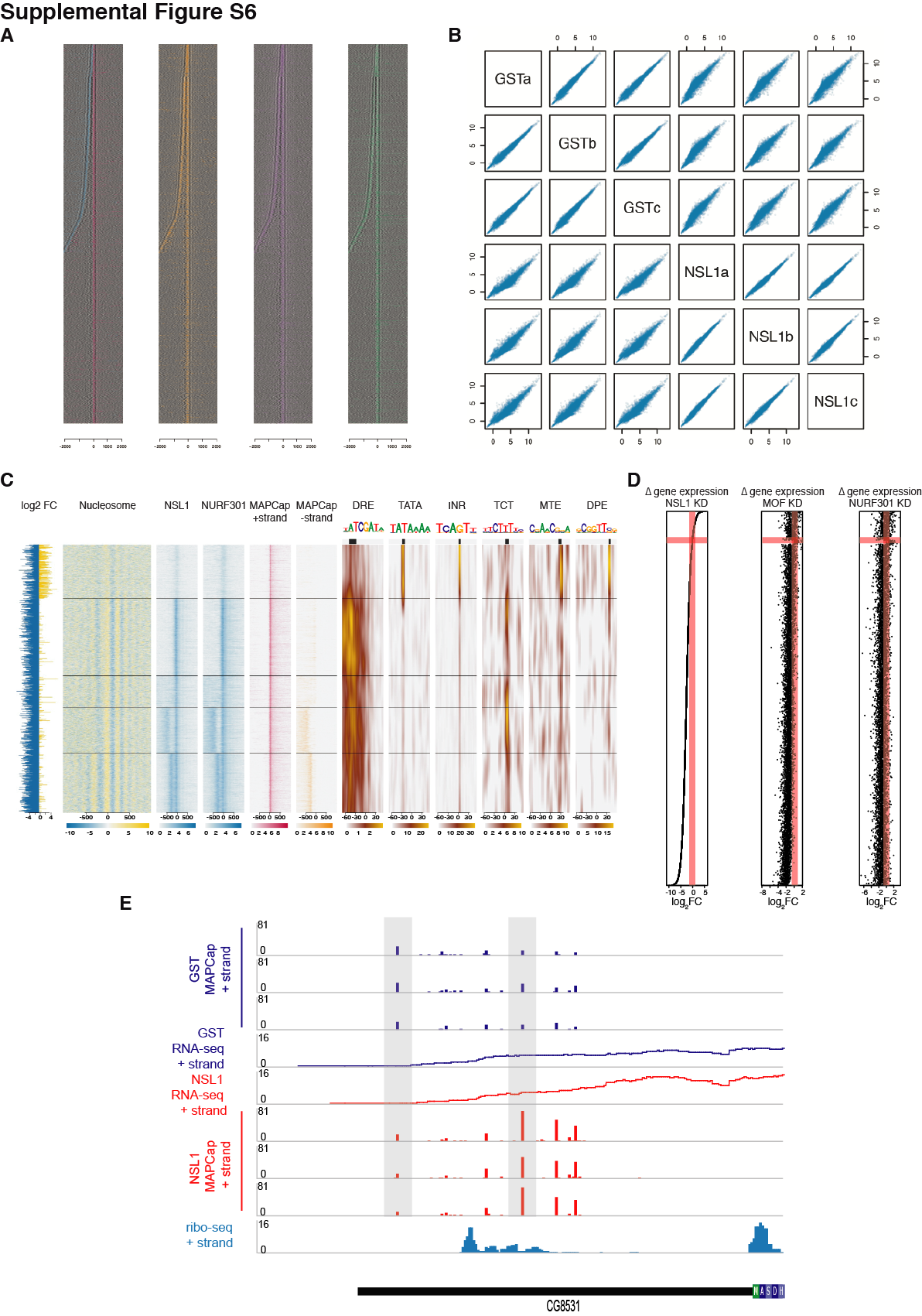


**Supplemental Figure S6**

(A) Heatmaps showing the overlap between TSS positions (MAPCap), NSL1 (orange), NURF301 (purple), Pol II (Rpb3, green) and nucleosome pattern (black).

(B) Scatter plots showing correlation between triplicates of the MAPCap data. Shown are the normalized and regularized log2 count values as computed by the rlog function of DESeq2. Triplicates of the GST and NSL1 samples show good correlation.

(C) Dominant MAPCap TSSs are clustered into five groups using the k-means clustering algorithm using matrices containing the ChIP-seq signals of NSL1 and NURF301, respectively. Each row of these matrices corresponds to a MAPCap TSS and the values to the ChIP-seq log_2_ enrichments in 143 29 base pair windows around the MapCap TSS. For these five groups of TSSs, we show (from left to right) tracks showing changes in gene expression upon NSL1 depletion. Blue denotes gene downregulation, yellow denotes gene upregulation. (1^st^ heatmap) changes in nucleosome signal upon NSL1 depletion, yellow indicates gain and blue indicate loss in signal. (2^nd^- 3^rd^ heatmap) ChIP-seq signal of NSL1 and NURF301. (4^th^-5^th^ heatmap) MAPCap data showing the position of TSS on plus (pink) and minus (orange) strands (6^th^ – 11^th^ heatmap) motif enrichment analysis for *Drosophila* core promoter motifs. The color indicates the enrichment.

(D) Scatter plots showing changes in gene expression in NSL1, NURF301 or MOF depleted cells. Genes are ordered according to the gene expression changes in NSL1 KD (leftmost).

(E) Representative example showing MAPCap, RNA-seq data for control and NSL1 KD samples, and ribo-seq data for wild type cells. Ribo-seq data shows upstream ORF in the 5’UTR region, where expression is reduced in NSL1 KD due to shift in TSS selection.


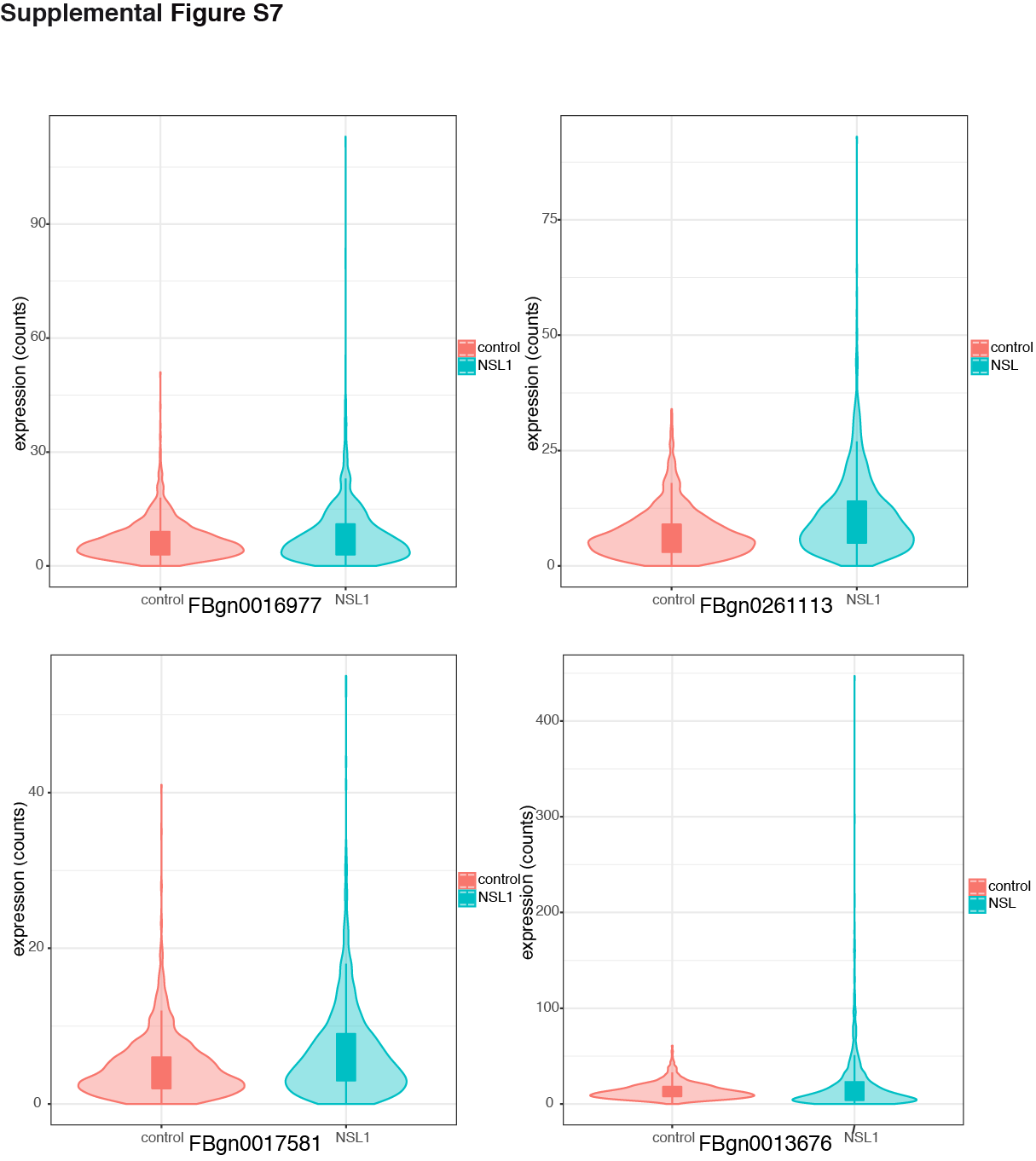


**Supplemental Figure S7**

Violin plots showing representative examples of changes in expression noise between control (red) and NSL1 KD (green). The lower and upper hinges of the boxplot correspond to the first and third quartiles while length of the whiskers represents 1.5 times the interquartile range.

| T7 MOF F | TTA ATA CGA CTC ACT ATA GGG AGA ATG TCT GAA GCG GAG CTG GAA CAG |
| --- | --- |
| T7 MOF R | TTA ATA CGA CTC ACT ATA GGG AGA CGAAGTCGTCAATGTTGGAACCAC TGCC |
| T7 NSL1 F | TTA ATA CGA CTC ACT ATA GGG AGA ATGGCCCCAGCGCTCACA |
| T7 NSL1 R | TTA ATA CGA CTC ACT ATA GGG AGA TGAACTTGTGGCCACTGCC |
| T7 EGFP F | TTA ATA CGA CTC ACT ATA GGG AGG ATGGTGAGCAAGG |
| T7 EGFP R | TTA ATA CGA CTC ACT ATA GGG AGG ATCGCGCTTCTCG |
| T7 NSL3 F | TTA ATA CGA CTC ACT ATA GGG AGA TCCTTGGCGACTACCTCATC |
| T7 NSL3 R | TTA ATA CGA CTC ACT ATA GGG AGA GTACCATTTCGGCCCCTAGTG |
| T7 NSL2 F | TTA ATA CGA CTC ACT ATA GGG AGA ACAGCGAGCTGGTCAAGATTAAG |
| T7 NSL2 R | TTA ATA CGA CTC ACT ATA GGG AGA TGCTCGAAGCAGAGGGTCGT |
| T7 WDS F | TTA ATA CGA CTC ACT ATA GGG AGA CCTGGAGCTCAGACTCGCGAC |
| T7 WDS R | TTA ATA CGA CTC ACT ATA GGG AGA GAATGGGCGGGTAGAGTCTTCAG |
| T7 Ebx F | TTA ATA CGA CTC ACT ATA GGG AGA CGAGCACTGATACTGCCAAA |
| T7 Ebx R | TTA ATA CGA CTC ACT ATA GGG AGA TACCTCCTTAATGATGCGGC |
| T7 ISWI F | TTA ATA CGA CTC ACT ATA GGG AGA ACGAGACGACTTCAGATGCG |
| T7 ISWI R | TTA ATA CGA CTC ACT ATA GGG AGA CCTTGTCTTTGATCTTCTTGGG |
| T7 Ino80 F | TTA ATA CGA CTC ACT ATA GGG AGA AGCGACAAAGTGTCAAAGCA |
| T7 Ino80 R | TTA ATA CGA CTC ACT ATA GGG AGA GCGGCCTTTTTGTAGTAGTTC |
| T7 MBDR F | TTA ATA CGA CTC ACT ATA GGG AGA CGCTGGCCACGTTTATTAAG |
| T7 MBDR R | TTA ATA CGA CTC ACT ATA GGG AGA TTGAAGAGAAAAAGCTTGTACGG |
| T7 GST F | TTA ATA CGA CTC ACT ATA GGG AGA TATCAATTTGTGGGATAGCT |
| T7 GST R | TTA ATA CGA CTC ACT ATA GGG AGA TTTTGGATATTAGATACGGT |
| T7 BRM F | TTA ATA CGA CTC ACT ATA GGG AGA GTGCCACCGAGAAGCTAGAG |
| T7 BRM R | TTA ATA CGA CTC ACT ATA GGG AGA TCATCCTCGGCCATCAGAC |
| T7 CHD3 F | TTA ATA CGA CTC ACT ATA GGG AGA CCGAAAACCAATGCCAAG |
| T7 CHD3 R | TTA ATA CGA CTC ACT ATA GGG AGA CCAGCGCCATGACAATATC |

**Supplemental Table S4**

List of primers used for amplification of dsRNA for knockdowns.

| Cg6506-pro-L | GCCGATGTTTACCGACAATC |
| --- | --- |
| CG6506-pro-R | CATGGTTGGTTATCGGGACT |
| CG6506-Mid-L | ATCCGTGCCTAATGATACCG |
| Cg6506-Mid-R | ACGGTTGGTGTGAACCAAAT |
| CG6506-end-L | ACAGTCAGCTCCCAGCAGAT |
| Cg6506-end-R | AAAGTGGCGTGAAAGTTGCT |
| Sec5-pro-L | G CTGCTCAGCAAGGAGACTT |
| Sec5-Pro-R | C GGACGAGCATAAAAAGAGC |
| Sec5-mid-L | G AACTCCCATTGGCGATAAA |
| Sec5-mid-R | A AATGTCTGGCGAAATGTCC |
| Sec5-end-L | A TCAACGGCTTCATCTTTCG |
| Sec5-end-R | G CGTTTTCTTCCATTTTCCA |
| OdsH-Pro-L | C CC ATT TTT CCC ACT GAC TG |
| OdsH-Pro-R | G GC GCG TAC AAA TGA AAA AT |
| OdsH-Mid-L | A AG ATC CGC TAA GCG ATG AA |
| OdsH-Mid-R | G CC AGG AGT TGA AGT TGG TC |
| OdsH-End-L | A GG CTC TCG TGG GGT AAA AT |
| OdsH-End-R | G AG CTC ACC GAT TTG TTT CC |
| Cg15011-pro-L | CAGCCCTGGTATTCGATGTT |
| CG15011-pro-R | CTCATCTTGGATCGGATCGT |
| CG15011-Mid-L | CCTGCCACAAGGAACACTTT |
| Cg15011-Mid-R | AGCTGCAACAAGCACAAATG |
| CG15011-end-L | ACACGGTGTTCTTCCAGTCC |
| Cg15011-end-R | CGCTAAGGAACGTCGAAATC |
| Cg4406-pro-L | CTGCTCGATAGCACGCAGT |
| CG4406-pro-R | TATCGACGGTCACACTGCTC |
| CG4406-Mid-L | CCTGGAACTTGAGGAATCCA |
| Cg4406-Mid-R | GGCAGCAATGTGCTCATCTA |
| CG4406-end-L | AGCTCGGAAGGAAACTGTGA |
| Cg4406-end-R | GTGACCAAAAAGCCCTTCAA |
| CG14872-Pro-L | AATCGAGACATTCAGGCACTC |
| CG14872-Pro-R | TTCCCACACTGAAAAATCCA |
| CG14872-Mid-L | AAGAGCTTGAACAGCGGAAC |
| CG14872-Mid-R | GATACGCAAACCGGCATC |
| CG14872-End-L | TCACGCTCTAAAACCCCAGA |
| CG14872-End-R | CAGTACGGCATGGGCAAC |
| Nup160-Pro-L | TAGCAAGATGCCCACATCCA |
| Nup160-Pro-R | ATTTTCACCTCGATCCACTCC |
| Nup160-Mid-L | AATGGGAACGATCAGACCAC |
| Nup160-Mid-R | CAGCGACAAACTGTCATGCT |
| Nup160-End-L | AAAATCATCCGTCATGTCCAG |
| Nup160-End-R | GCGCCAAGCACATTTTTC |
| Patj-Pro-L | G AGTGCATAGGAGAGGGTAAACA |
| Patj-Pro-R | G TGGCGTTGGCACACTTT |
| Patj-Mid-L | C GTCGGTCACCACAATGA |
| Patj-Mid-R | T TATCCGCCAAGGGTACAAC |
| Patj-End-L | A CGCGGTTGCTAACTAATGG |
| Patj-End-R | A CTTCTGGCATCGTTTCTGAC |
| tho2-Pro-L | C CTCGGATCAGGTGGTACA |
| tho2-Pro-R | G TCACACTGGCGGAACTAACT |
| tho2-Mid-L | G GCCACATCCGTGTTTATGT |
| tho2-Mid-R | G CCAAGACACACTCGTCCA |
| tho2-End-L | G CTTCACAATGCACGGAAC |
| tho2-End-R | G AGGAGCGGCAGTACATCA |
| Ent2-Pro-L | C GTAACGGCACCCCTCAA |
| Ent2-Pro-R | A CCGCACCGCACTACAAG |
| Ent2-Mid-L | C CGCCATCCTAGTGCTGCT |
| Ent2-Mid-R | G CTGCTCCGGCTAATGGT |
| Ent2-End-L | T CTCGTATCTGGGACCATTTT |
| Ent2-End-R | T CCCGGAACTGGTATTGAG |
| Bap170-Pro-L | CCTGCTCGTGAATGCAACT |
| Bap170-Pro-R | GTGGCGTGAATGGGAAAC |
| Bap170-Mid-L | ACCCCCAGCATTGTTCCT |
| Bap170-Mid-R | CTTTCCTCAGACGCCACTTC |
| Bap170-End-L | ATGAACCGACACACGACTGA |
| Bap170-End-R | GCCGTAGCCGAGTAGGTGA |
| Incenp-Pro-L | GTTCTTTCCCTTACCATTTTCC |
| Incenp-Pro-R | GTTCCCGCCACTACCATCT |
| Incenp-Mid-L | GAGGACGAGTCGGTGGAG |
| Incenp-Mid-R | TTGAAAAGCTCATGTGTACGG |
| Incenp-End-L | GCCACGTAAGGGGAGAGG |
| Incenp-End-R | GTTCGGGAATATCTGCTTTAGG |
| ns4-Pro-L | GAGATGCCAACTTGTAGGTGATT |
| ns4-Pro-R | AA ATACATGCAGAGACAGGAGGT |
| ns4-Mid-L | GC AAGGTGGTCAGCGTTAGT |
| ns4-Mid-R | GA CTAGACCGGGACAATCACA |
| ns4-End-L | GA CAGCGAGGATGAAGACGA |
| ns4-End-R | CA GCAGAGCAAACACGTTCC |
| aur-Pro-L | CG CTTCACAATTCAAACGA |
| aur-Pro-R | AC AGGCACTCGACAACTAACA |
| aur-Mid-L | GG ACATCAAGCCGGAGAA |
| aur-Mid-R | AT TTCGGGTGGCAAGTAGTC |
| RecQ4-Pro-L | GTCTGGCAAGGTCGGTGA |
| RecQ4-Pro-R | ATGTCGGCGGCTGTCTTT |
| RecQ4-Mid-L | CGGAGAAAAAGCCGAGGA |
| RecQ4-Mid-R | GTAATATCTCCCGCCTCCAA |
| p5cr-Pro-L | C ACACCAAAGCTCAGAGGAGT |
| p5cr-Pro-R | C CGATTGCATGGGCGTAG |
| p5cr-Mid-L | G CGAGGGCTGCACTGTTT |
| p5cr-Mid-R | T GGACTCGGGCACCTGTT |
| p5cr-End-L | A TGTAATCCCCCGGAACA |
| p5cr-End-R | G CAAGAAGGATCGGGAATAA |
| Ns4-Pro-L2 | C TGCACCTCCTGTCTCTGCATGT |
| Ns4-Pro-R2 | A GAGCACCGGGTTGTCGAGC |
| Ns4-Mid-L2 | C ACGCCCCACGAGCACGTTA |
| Ns4-Mid-R2 | C GCTGACCACCTTGCGTCCC |
| Ns4-End-L2 | A CAACACCAAGCTATGGAGGCAGA |
| Ns4-End-R2 | C TCGCTAGACGAAAGACTCGGCG |

**Supplemental Table S5**

List of primers used for quantitative PCRs

**Materials and Methods**

**Drosophila cell cultures**

S2 cells and Kc cells were cultured in Schneider’s medium (Gibco) supplemented with 10% FCS and 0.05% Pluronic F-68 (Sigma-Aldrich). The cultures were maintained on shaking incubators at 27 ºC, at a speed of 80 rpm. The cells were kept at a density of 1-16 million/ml.

***Drosophila* rearing conditions and genetics**

Unless otherwise specified, flies were reared on a standard cornmeal fly medium at 25 °C, 70% relative humidity and 12 hr dark/12 hr light cycle. The following stocks were obtained from the Bloomington stock centre or kindly donated:

y1 w*; P{tubP-GAL4}LL7/TM3, Sb1 (Bloomington stock #5138)

w1118;P{da‐GAL4.w‐}3 (Bloomington stock #8641)

y1 w1118; P{lacW}nsl1j2E5/TM3, Sb1 (Bloomington stock #12304)

w1118; P{GD13852}v24248 (UAS-nsl3dsRNAi) (VDRC stock #24248)

y1 v1; P{TRiP.JF01299}attP2 (UAS-Nurf-38dsRNAi) (Bloomington stock #31341)

y1 sc* v1; P{TRiP.HMS00065}attP2 (UAS-Nurf-301dsRNAi) (Bloomington stock #33658)

y1 sc* v1; P{TRiP.HMS00628}attP2/TM3, Sb1 (UAS-IswidsRNAi) (Bloomington stock #32845)

y1 v1; P{TRiP.HMC03329}attP40 (UAS-Mi-2dsRNAi) (Bloomington stock #51774)

y1 w1118; nsl1e(nos)1/TM6B, Tb (Yu et al. 2010)

P{w^+^,UAS_GAL_hsp70:ISWI^K159R^}11–4  (UAS-Iswi^K159R^) (Deuring et al. 2000)

All lines used in this study were generated via standard genetic crosses from the above listed stocks. To assess the genetic interaction between nsl1 and nsl3, Nurf-38 and Nurf-301, w;; daGal4/TM6Tb and w;; daGal4, nsl1j2E5/TM6B, Tb virgin females were crossed with UAS-nsl3dsRNAi, UAS-Nurf-38 and Nurf-301 males and flies were allowed to develop at 25 °C or 29 °C in the latter case. To determine the relative viability, male and female adult flies from at least three independent crosses were counted every other day for a period of 10 days from the start of the eclosion. The total number of non-Tb males and females was divided by the total number of Tb males and females respectively, which were used as an internal control with 100% viability.

To assess the genetic interaction between nsl1 and Iswi and Mi-2, w;; tubGal4/TM6B, Tb, Ubi-GFP and w;; tubGal4, nsl1e(nos)1/TM6B, Tb, Ubi-GFP virgin females were crossed to UAS-Iswi-dsRNAi/TM6B, Tb, Ubi-GFP and UAS-Mi-2dsRNAi males and flies were allowed to lay eggs on yeast-supplemented apple juice plates. 0-6 hour collections were used 24 hrs later to separate fluorescent from non-fluorescent larvae under a fluorescent stereomicroscope and at least 3 plates with 100 larvae/plate were monitored and the rate of pupariation/number of pupae determined over a period of 3 weeks.

It is important to note that although the used heterozygous nsl1 null mutant alleles do not affect the viability of the flies, it has been previously reported that nsl1e(nos)1/+ has reduced NSL1 function rendering it amenable to genetic interaction analysis (Yu et al. 2010). When a strong ubiquitous driver, tubGal4, was used for dsRNAi induction at 25°C, this resulted in 100 % lethality (data not shown) recapitulating the loss-of-function phenotype previously reported for nsl3, Nurf-38, Nurf-301, Iswi and Mi-2.

The used combinations of daGal4 or tubGal4 and 29°C, 25°C or 18°C aimed at partial lethality or sensitized genetic background allowing for enhancers or suppressors of the lethality phenotype to be identified.

For Fig. 2D, in order to minimize background effects (Chari and Dworkin 2013), we chose a set of *UAS-RNAi* lines (Flockhart et al. 2006; Perkins et al. 2015; Hu et al. 2017) that efficiently silence the target genes, leading to 100% lethality, and established loss-of-function mutants of these genes. We used either a weaker Gal4 driver (*da-Gal4*), or lower temperatures, to establish conditions of partial lethality.

To further control for biases of the approach, in addition to the RNAi-based experiments we also used a previously characterized *Iswi^K159R^* dominant-negative allele (Deuring et al. 2000). This K159R substitution eliminates the ATPase and the chromatin remodeling activities of ISWI (Corona et al. 1999) without affecting neither the stability of the ISWI protein nor its incorporation into high molecular weight complexes.

**Preparation of nuclear extract from S2 cells**

Wild type S2 cells or S2 cells stably expressing the NSL complex members were harvested and washed with ice-cold PBS. The volume of the cell pellet was estimated. The cells were re-suspended and incubated in 5 pellet cell volumes (PCV) of cold hypotonic buffer (10 mM HEPES pH 7.9, 1.5 mM MgCl_2_, 10 mM KCl and Roche complete Protease Inhibitor Cocktail) on ice for 15 minutes. NP-40 was then added to the hypotonic buffer to a final concentration of 1% and the cells were immediately vortexed for 30 seconds. The cells were centrifuged at 2000g for 5 minutes. The supernatant was kept as cytoplasmic extract while the pellet was kept as nuclei. The nuclei were re-suspended and washed with 5 PCV of cold isotonic solution (25 mM HEPES pH 7.6, 2 mM MgCl_2_, 3 mM CaCl_2_, 300 mM sucrose and Roche complete Protease Inhibitor Cocktail). The washed nuclei were carefully re-suspended in 5 PCV of HEMGT 250 buffer (25 mM HEPES pH 7.4, 0.1 mM EDTA, 250 mM NaCl, 1 mM MgCl_2_, 0.1% Triton X- 100, 10% glycerol and Roche complete Protease Inhibitor Cocktail) and rotated for 2 hours at 4 ºC. The nuclei were finally centrifuged at 18000g for 30 minutes. The resulting supernatant contained the nuclear extracts.

**Co-immunoprecipitation**

The nuclear extract prepared as aforementioned was diluted with an extraction buffer without salt to lower the NaCl concentration from 250 mM down to 150 mM. If the extract went through a freeze-thaw process, it would always be further centrifuged at 14000 g for 15 minutes before being used in immunoprecipitation experiments. 200 ul of the extract was used for immunoprecipitation. HEMGT 150 buffer (25 mM HEPES pH 7.4, 0.1 mM EDTA, 150 mM NaCl, 1 mM MgCl_2_, 0.1% Triton X-100, 10% glycerol and Roche complete Protease Inhibitor Cocktail) was used to increase the volume to 600 ul. Protein A/ protein G beads (Protein A/G Sepharose 4 Fast Flow from GE Healthcare) was used to pre-clear the extract. Before use, all beads were pre-equilibrated with HEMGT 150. Extracts were incubated with 2 to 5 ul of the antiserum (depending on the strength of the antibody) for 4 hours to overnight at 4 ºC. 5 ul of rabbit/rat IgG or pre-immune serum was used in the control experiments. 40 ul of protein A/ protein G slurry was added and the tubes were mixed on a rotator at 4 ºC for another hour. The beads were then spun down at 1000 g for 1 minute. 6 HEMGT-150 washes with 1 minute of rotation in 4 ºC were performed. Finally, the beads were boiled in 100 ul of 2X Laemmli Sample Buffer (Rotiload, Roth) for 5 minutes. For immunoprecipitated samples, 18 mM of iodoacetamide was added to block reduced cysteine residues and ensure complete denaturation of the eluted antibodies. The samples were incubated for 30 minutes in the dark. 15 ul of the samples was analyzed using SDS PAGE. Anti-ISWI, anti-BRM and anti-Mi-2 (from Dr. Peter Verrijzer), anti-CHD3 antibody (from Dr. Alexander Brehm), anti-NURF38 (from Dr. Paul Badenhorst) are used for western blot analysis. Anti-NSL complex antibodies (Mendjan et al. 2006) and anti-NURF301 are the same antibodies as in the ChIP experiments.

For co-immunoprecipitation experiments using stable cell lines, anti-FLAG M2 agarose beads (Sigma) were used. The beads were mixed with the extract for 2 hours at 4 ºC on a rotating wheel.

**Knockdown experiments in S2 cells**

Since the double-stranded RNAs were targeting the exons of the genes, genomic DNA was used as a template in the PCR amplification. For double-stranded RNAs against GST, the plasmid pET-41a (milipore), which contains a GST tag, was used as a template. The amplification was done for 35 cycles using Phusion High Fidelity Polymerase according to the manufacturer’s recommendations. The DNA was loaded on an agarose gel to ensure that a specific PCR product was amplified. When multiple bands appeared on the gel, a different primer pair was used in the amplification, or the DNA was purified by gel extraction. The DNA could also be purified with QIAquick PCR Purification Kit (Qiagen) if the PCR reactions were confirmed to produce one specific product. Double-stranded RNAs were generated using the Ribomax Large Scale T7 *in vitro* transcription system (Promega) according to the manufacturer’s instructions. The RNAs were then purified using the MEGAclear (Ambion) and eluted in nuclease-free water (Invitrogen). To ensure that the two strands of the RNAs annealed perfectly to each other, the RNAs were heated to 65 ºC for 10 minutes and allowed to cool down slowly to room temperature.

**Luciferase assays**

100 ul of cells at 1 million cells per ml was plated on 96 wells plates. Cells in each well were transfected with a plasmid mixture of (1) 200ng of pG5luc, which contains the firefly luciferase gene whose expression is controlled by UAS sequences (2) 2ng of pRL-hsp70, which contains a constitutively expressed Renilla luciferase gene and (3) 50ng of pAc5.1 vector containing either Gal4DBD-tagged NSL3 protein, or Gal4DBD alone. The Renilla luciferase acts as a control for normalization of transfection and cell number variation. Transfections were carried out with X-tremeGENE DNA Transfection Reagents (Roche). After 2 days of incubation, cells were lysed (Dual-Luciferase Kit, Promega) and luminescence was measured by using Mithras plate reader (Berthold). To test the Gal4-NSL3 activity upon depletion of the NSL complex and chromatin remodelers, double stranded RNAs against the proteins of interest were transfected to the cells on 96 well plates. The knockdown experiments were carried out as described in the previous section. 2 days after the RNA transfection, the luciferase gene-containing plasmids were transfected to the cells as described above.

**Chromatin Immunoprecipitation (ChIP)**

50 million S2 cells were harvested and washed with PBS. The cell was resuspended in 1ml of crosslinking solution (50 mM HEPES, 100 mM NaCl, 1 mM EDTA, 0.5 mM EGTA), 37% formaldehyde was added to a final concentration of 1.8%. The tube was rotated at room temperature for 10 minutes and the reaction was quenched with 2.5 M glycine at a final concentration of 125 mM. The cells were spun down immediately after addition of glycine, and the solution was replaced with fresh PBS with 125 mM glycine. The cells were resuspended and rotated for 5 minutes at room temperature. All the steps that follow were carried out either at 4 ºC or on ice. The cells were pelleted by centrifugation at 2000rpm for 5 minutes. The cells were washed three times with Paro 1 (10 mM Tris pH 8.0, 10 mM EDTA, 0.5 mM EGTA, 0.25% TritonX-100), four times with Paro 2 (10 mM Tris pH 8.0, 200 mM NaCl, 1 mM EDTA, 0.5 mM EGTA) and twice with RIPA (140 mM NaCl, 25 mM HEPES pH 7.5, 1 mM EDTA, 1% TritonX-100, 0.1% SDS, 0.1% DOC). For each washing step, cells were resuspended in the mentioned buffers and rotated in the cold room for 5 minutes, before they were spun down again. After all the washing steps, cells were resuspended in 500 ul RIPA and sonicated using a Branson sonificator followed by Covaris sonication. After sonication, an aliquot of the chromatin was decrosslinked, treated with Protease K/RNaseA and the DNA was purified. The DNA was analyzed with agarose gel to ensure that the chromatin was sheared into fragments with average length of 200 bp. The number of cycles can be adjusted to achieve the desired fragment length.

For NSL1 and NURF301 MNase-ChIP-seq, we used MNase for chromatin shearing. Cells were washed as above described and suspended in MNase digestion buffer. A small aliquot of the samples is used to determine the optimal MNase concentration (20-200 U/ml tested) to obtain the desired fragment size. Digestion is performed in room temperature for 20 minutes and finally quenched by addition of 1 mM EDTA and 2X RIPA buffer.

Chromatin was centrifuged at 14000 rpm for 30 minutes and the pellet was discarded. The supernatant was incubated with Protein A/ Protein G sepharose beads and rotated in the cold room for 30 minutes to preclear the chromatin. The concentration of the chromatin was determined with a NanoDrop 1000 Spectrophotometer (thermo). For each IP, 10 ug of chromatin was diluted in RIPA to fill up the volume to 500 ul. 3 ul of antibody was added to the chromatin and rotated overnight at 4 ºC. Anti-NURF301 (Novus Biologicals‎) and anti-NSL1 (Mendjan et al. 2006) antibodies are used. Protein A/G sepharose was blocked with 0.1% BSA and 1 mg/ml of short dsDNA (15 bp) for 1 hour. 20 ul of sepharose was added to the chromatin/antibody mixture and incubated for 3 hours at 4 ºC. Protein A sepharose was used if the antibodies came from rabbit or guinea pig; protein G agarose was used if the antibodies came from rat. The sepharose bead was then washed six times for 5 minutes in RIPA buffer, once in LiCl buffer (0.25 M LiCl, 10 mM Tris pH 8, 1 mM EDTA, 0.5% NP-40, 0.5% DOC), and two times in TE buffer. The bead was incubated at 65 ºC overnight in TE buffer to reverse the crosslink. The sample was then treated with RNaseA (0.2 mg/ml) for 30 minutes at 37 ºC, and with Proteinase K (0.05 mg/ml) for 2 hours at 50 ºC. Finally, DNA was purified using Minelute columns (Qiagen) and eluted in nuclease-free water. This protocol generated enough material for quantitative real time PCR analysis. For ChIP-seq analysis, the same protocol was scaled up until 10 ng of DNA was achieved.

The eluted DNA from the ChIP protocol was analyzed by qPCR using SYBR Green Master Mix (Roche) and ABI7500 real-time PCR thermocycler (Applied Biosystems, Inc.). For ChIP samples, 10% and 1% INPUT materials were included in the analysis. Efficiency of the ChIP (as input recovery) was determined as the amount of immunoprecipitated DNA relative to input DNA.

**Micrococcal nuclease digestion followed by sequencing (MNase-seq)**

50 million S2 cells were harvested and washed with PBS. Cells were counted very carefully to ensure that the same number of cells was collected for each sample. The cells were resuspended in 1 ml of crosslinking solution (50 mM HEPES, 100 mM NaCl, 1 mM EDTA, 0.5 mM EGTA) and 37% formaldehyde was added to a final concentration of 1%. The tube was rotated at room temperature for 10 minutes and the reaction was quenched with 2.5 M glycine at a final concentration of 125 mM. The cells were spun down immediately after addition of glycine and the solution was replaced with fresh PBS that contained 125 mM glycine. The cells were resuspended and rotated at room temperature for 5 minutes. The cells were pelleted into equal aliquots by centrifugation with 2000 rpm for 5 minutes. Aliquots were then snap frozen in liquid nitrogen.

Cells were permeabilized with NP-40 as described in the previous section. The chromatin was digested with various amount of MNase at 25 ºC for 10 minutes. Digested chromatin was then analyzed on an agarose gel to reveal the digestion pattern. We aimed to achieve a slightly under-digested condition with di- and tri-nucleosomes still visible. In addition, the digestion pattern of each sample needed to be exactly the same. After the optimal amount of MNase was determined for each individual experiment, a new aliquot of the cells was again digested with MNase at 25 ºC for 10 minutes. The reaction was stopped by addition of EDTA, NaCl and SDS to an end concentration of 20 mM, 150 mM and 1% respectively. The samples were placed on ice during handling to ensure that the MNase digestion was halted completely. The chromatin was then de-crosslinked overnight at 65 ºC overnight in TE buffer. The sample was treated with RNaseA (0.2 mg/ml) for 30 minutes at 37 ºC, and with Proteinase K (0.05 mg/ml) for 2 hours at 50 ºC. Finally, DNA was purified using Minelute columns (Qiagen) and eluted in nuclease-free water.

The eluted DNA was then analyzed with the E-Gel Pre-cast Agarose Gels system (Invitrogen). Bands at positions corresponding to 150 bp, 300 bp, 450 bp, represented mono- di and tri-nucleosomes, respectively. The 150 bp fragment was purified from the E-gel according to manufacturers’ specifications and used to generate the library for the Illumina paired-end sequencing. To minimize the effect of PCR artefacts, only 3 cycles of PCR were performed in the library generation procedure.

## MNase-seq analysis

### MNase-seq read mapping

The paired-end reads were aligned using Bowtie2 (version 2.0.5; (Langmead and Salzberg 2012)) or with STAR (version 2.5.1b; (Dobin et al. 2013)) against the *Drosophila melanogaster* reference genome (version dm3, UCSC). Default parameters were used, except for the STAR alignment, where we set the option alignIntronMax to 1. In case of Bowtie2 resulting BAM files were sorted using samtools (Li et al. 2009), while this step was omitted for STAR alignments as they were already sorted. The sorted BAM files were indexed using samtools.

### Estimation of the nucleosome signal

To get an unbiased estimate of the nucleosome signal we counted the 5’ starting positions of the reads separated by their orientation, i.e. plus- and minus-strand alignments. This results in two vectors and with components and, where indicates the position and corresponds to the counts for plus-strand reads and for minus-strand reads. For all positions where we calculated , while for positions where .

For a well-positioned nucleosome at its dyad position we expect many more plus-strand reads than minus- strand reads at its left border at position and vice versa many more minus strand reads than plus-strand reads at its right border at position. To account for the inprecise mapping of the nucleosome borders by MNase-seq, we defined a function for and zero otherwise. This function was convolved against the vector to obtain the nucleosome signal at the nucleosome dyad position . This nucleosome signal is between -74 and +74, where a negative value indicates depletion and positive signal enrichment. Note, that this procedure yields a normalized nucleosome signal, which is independent on library size.

## ChIP-seq analysis

### ChIP-seq read mapping

The ChIP-seq reads were aligned either with Bowtie2 or STAR and processed as outlined in the MNase-seq read mapping section.

### Peak calling

Peaks were called using the Bioconductor package normR (<https://www.bioconductor.org/packages/normr>; (Kinkley et al. 2016). Reads/fragments were counted using the Bioconductor package bamsignals (<https://www.bioconductor.org/packages/bamsignals>).

For single-end ChIP libraries the fragment size was estimated by convolving the vector of counts of minus strand reads against the corresponding vector of plus strand reads. The fragment size was determined by choosing the distance for which was maximal.

For paired-end ChIP and input libraries we counted the number of fragment midpoints falling into non-overlapping 200 base pair bins along the genome. For single-end ChIP and input libraries the 5’ positions of the reads were shifted by half the fragment size to the right for plus strand and to the left for minus strand reads. For each ChIP experiment a normR analysis was performed using the function enrichR, where the count vectors for the ChIP and the corresponding input control served as input.

Normalized log_2_ Chip over control were calculated by where corresponds to the number of ChIP fragments/reads in a bin of size , x to the number of control fragments/reads in the same bin. to the average number of ChIP fragments/reads in 200 base pair bins that had a qvalue > 0.1 divided by 200 and multiplied by , and to the average number of control fragments/reads in 200 base pair bins that had a qvalue > 0.1 divided by 200 and multiplied by .

## RNA-seq analysis

### RNA-seq read mapping

The paired-end RNA-seq reads were aligned using STAR (version 2.5.1b) against the *Drosophila melanogaster* reference genome (version dm3, UCSC) plus the *Saccharomyces cerevisiae* genome (release R64-1-1, ENSEMBL) including the corresponding gene annotations from both species.

### DEseq2 analysis

Standard RNA-seq analyses are not able to detect global shifts in gene expression. We expected that a NSL1 knockdown may lead to a global downregulation of almost every expressed gene. In such a scenario, genes that are un- or only mildly affect by the NSL1 knockdown would appear upregulated. To mitigate this problem, we spiked yeast RNA into the RNA preparations according to the cell number, which had been estimated by qPCR. The yeast RNA serves as external control to normalize the RNA-seq data, because the abundance of yeast RNAs remains unchanged. Thus, after mapping to the combined *Drosophila* and yeast reference genomes, we obtained the read counts for the 16 yeast chromosomes. And used these counts to calculate size factors using the DESeq2 (Love et al. 2014) function estimateSizeFactorsForMatrix using triplicates for control, MOF, NSL1 and NURF knockdowns.

For each condition and replicate we counted reads mapping to annotated exon ranges per gene, where overlapping exons were merged. We counted only the first mate to retain the strand information.

A preliminary principal component analysis on the rlog transformed counts revealed that two samples, one control and one NSL1 sample, were outliers, i.e. did not cluster with their respective conditions. These were removed prior to any downstream analysis.

With the remaining 10 samples we performed a DESeq2 analysis, where we manually set the size factors to the ones estimated by the yeast spike ins. We used the condition, i.e. either control, MOF, NSL1 or NURF knockdown, as explanatory variables. After the DESeq analysis we obtained results by contrasting MOF, NSL1 or NURF against the control.

## gDNA Analysis

**Genomic DNA immunoprecipitation followed by sequencing**

Genomic DIP–seq experiments were performed as described (Gossett and Lieb 2008) with few modifications. The pellet from 1 × 10^8^ S2 cells was suspended in 1 ml of lysis buffer (10 mM Tris pH 8, 25 mM EDTA, 100 mM NaCl, 0.1% SDS) and treated with protease K overnight at 55 °C. The DNA is tested with Bradford assay and protein gel to ensure that no proteins remain. DNA is sonicated with Covaris sonicator (power 175 W, duty factor 10%, cycles per burst 200, 250 s) to obtain fragment size average of 200-bp. DNA was purified with the DNA Clean & Concentrator (Zymo).

FLAG-NSL3 and FLAG-GFP were expressed in baculovirus. The extract was prepared two days after infection. Details of the protocol are available upon request.

Proteins were purified using FLAG bead slurry (sigma). The beads were incubated with the extracts for 2 hours followed by 4 washes in HEMGT buffer (25 mM HEPES, pH 7.9, 12.5 mM MgCl2, 150 mM KCl, 10% glycerol). DIP-seq experiment was performed with 1μg of genomic DNA and 1.5 μg of FLAG-NSL3 or FLAG-GFP at 4 °C for 2 hours in 500 μl of DIP buffer (2 mM Tris-HCl pH 7.5, 100 mM KCl, 2 mM MgCl2, 10% glycerol, 10 μM ZnCl2). The FLAG bead was washed six times with 1ml of DIP buffer. Elution was performed with 3X FLAG peptide (100μg/ml, overnight) or digestion with proteinase K (1 h at 56 °C), DNA was purified with the DNA Clean & Concentrator (Zymo). The sequencing was performed using the Illumina HiSeq platform with a 75-bp paired-end kit.

### gDNA read mapping and peak calling

The paired-end gDNA-seq reads were aligned using STAR (version 2.5.1b) against the *Drosophila melanogaster* reference genome (version dm3, UCSC) and processed as outlined in the MNase-seq read mapping section.

Peak calling of the gDNA pulled down by GFP, MCRS2, NSL1 and NSL3 was performed as outlined in the ChIP-seq peak calling section using 50 base pair bins. As control served the input to the pull down experiments. After the identification of enriched bins, we merged directly adjacent bins and determined the maximal bin within the so-derived regions. The coordinates of these maximal bins per enriched region were referred to as peak center.

### Correlation of gDNA enrichment to sequence features

We computed the normalized and regularized log enrichment using the normR function getEnrichment with parameter standardize = FALSE (Kinkley et al. 2016). Using the peak centers as anchor points, we determined the AT content in 50 base pair bins in a window +/- 1,000 base pairs around these anchor points. We sorted the peaks by the average log enrichment +/- one bin around the peak center bin.

For the whole genome, we repeated the normR analysis using 200 base pair bins. For each of the 200 base pair bins, we calculated the AT content as well as the frequency of all 1,024 possible 5mers. We plotted the AT content in percent against the log enrichments for all bins which were not filtered out by the T-filter employed in normR and calculated the Pearson correlation coefficient.

To exclude the possibility that not the AT content but rather a certain AT-rich motif explains the *in vitro* binding of NSL3 we linearly regressed the *in vitro* NSL3 log enrichment over control against the AT content + the frequency of all possible 5mers. This effectly removes transitive, i.e. indirect correlations. For example, if the frequency of the 5mer AAAAA is correlated to the NSL3 log enrichment only because it is more likely to find such a 5mer in an AT-rich sequence then the partial correlation framework would reduce the information contained in the frequency AAAAA about the NSL3 log enrichment. However, if the frequency of AAAAA remains informative for the NSL3 log enrichment after accounting for its increased frequency in AT-rich sequences then this would be a statistical argument to propose that the AAAAA is preferentially bound by NSL3.

### Prediction of in vivo NSL3 MapCap TSS targets

We used the MapCap TSSs as anchor points to compute the NSL3 enrichment in 61 (30 upstream, 1 corresponding to the TSS, and 30 downstream) 29 base pair bins and the AT content in the same bins. We clustered the MapCap TSSs by the average NSL3 enrichment 11 bins around the TSS via kmeans into NSL3 bound and non-bound. Next, we assigned the value +1 for NSL3 bound TSSs and -1 for NSL3 non-bound TSS. The resulting vector was used as response variable for logistic regression. Because ~70% of the TSSs were NSL3 bound, we used a sampling strategy, were we sampled the same number of NSL3 bound and non-bound TSSs with replacement to get a balanced set. We then performed standard linear regression using 10-fold cross validation.

We performed a similar analysis with the DRE motif, where we used a window of -70 to -10 (i.e. 61 features) upstream of the TSS. This region should encompass the preferential location of the DRE motif.

To assess the goodness of fit, we randomly sampled class assignments, i.e. NSL3 bound and non-bound, with probability 0.687 for NSL3 bound and 0.313 for NSL3 non-bound 1,000,000 times. For each sample we calculated the number of correctly “predicted” class labels.

We also checked for the occurrence of the core promoter motifs: DRE, TATA, INR, MTE and DPE around the MapCap TSSs. We computed the PWM scores around and assigned a positive match to a binding site by requiring that the score is among the 1% highest scores along the genome.

## MapCap analysis

### MapCap read mapping

MapCap paired-end reads were mapped as outlined in the RNA-seq read mapping section. Prior to mapping the corresponding unique molecular identifier (UMI) was added to the read name to preserve it for downstream analysis.

### MapCap analysis

The MapCap approach aims at identifying 5’ cap positions at single base pair resolution. Due to the limited input material, an amplification of the library via PCR is necessary. The PCR leads to the problem that it remains unclear whether an accumulation of reads at a certain 5’ cap position is due to high expression or due to amplification. Furthermore, the PCR step introduces additional variance in the data, e.g. by differential amplification rates due to sequence composition and fragment length. To mitigate these problems a UMI was added to each fragment, such that PCR artifacts can be filtered out.

To use the UMI information the UMIs were extracted from the read name and appended as RX tag to the tag section of the BAM record by a custom perl script. PCR duplicates were removed using the Picard (version 2.5.0; <http://broadinstitute.github.io/picard/>) function MarkDuplicates by setting the options REMOVE_DUPLICATES=true and BARCODE_TAG=RX.

The paired-end MapCap reads can be used to assign a putative 5’ cap position to the corresponding gene. This can be achieved by mapping the second mate to the annotated exons and assigning the 5’ cap position to the corresponding gene. We used a custom perl script to perform these assignments whose output denotes the gene, chromosome, position, strand and count of 5’ caps, which we will refer to as transcriptional start sites (TSSs).

We constructed a union of all TSSs by requiring at least 2 reads per TSS and sample (control, NSL1 knockdowns in triplicates) using a custom perl script whose output denotes the gene, chromosome, position, strand and counts for all samples. To each gene we assigned one TSS, which had the maximal number of summed counts in the three control knockdown samples. From the identity of these TSSs we derived a count matrix, which served as input to a DESeq2 analysis as outlined in the RNA-seq/DESeq2 analysis section.

To identify genes which change their TSS usage, we performed the following analysis: First, we extracted the counts for each TSS of a gene for the control and NSL1 knockdown triplicate samples. Second, we retained only TSSs that were covered either in all three GST or NSL1 KD samples or in all six samples. Third, if more than one TSS remained we calculated the likelihood of two probabilistic models: the Null model, where we used a single multinomial distribution with parameters to model the TSSs counts per sample. We parametrized these “success” probabilities by , where denotes the counts for TSS in sample . The alternative model, where we used one multinomial distribution for the control samples and another one for the NSL1 knockdown samples with parameters and , which were parameterized by , where denotes the condition, control or NSL1.

The likelihood for the Null model for sample is and the likelihood for the alternative model for sample is . From these per sample likelihoods we computed the total log likelihood by for model .

Finally, we performed a log-likelihood ratio test with degrees of freedom. We adjust the so-computed p-values for multiple testing by the Benjamini and Hochberg correction.

**Riboseq analysis**

The riboseq data is downloaded from the Gene Expression Omnibus (Dunn et al. 2013). We downloaded the unmapped ribo-seq data, aligned them using Bowtie2 against the *Drosophila* melanogaster BDGP Release 5 (dm3) genome assembly and produced strand-specific coverage to be visualized using Integrative Genomics Viewer (IGV).

## scRNA-seq Analysis

**Sample preparation**

dsRNA-mediated RNAi is performed as described above. Single cells in the control and NSL1 KD samples were carefully diluted, re-suspended and filtered to obtain single cell suspension. The cells are then captured using the 10x genomics Chromium Single Cell 3’ Library and Gel Bead Kit v2, 4 rxns (10x Genomics, 120267) according to the manufacturer’s instructions (Single Cell 3’ Reagent Kits v2 User Guide, CG00052). Sample was loaded to a different lane on a chip (Chromium Single Cell A Chip Kit, 16 rxns, 1000009). After generation of nanoliter-scale Gel bead-in-EMulsions (GEMs), RNA contained in the GEMs were reverse transcribed in a Thermal Cycler (Bio Rad) ( 53°C for 45 min, 85°C for 5 min, hold at 4°C). Then, the single-strand cDNA was isolated and amplified. Finally, the amplified cDNA was fragmented, end-repaired, A-tailed and indexed using the Chromium i7 Multiplex Kit 96 rxns (120262). The clean-up steps during the library generation was performed with Ampure beads. These libraries were sequenced using the Illumina HiSeq platform with a 75-bp paired-end kit. Alignment and initial processing of sequencing data were performed with CellRanger package using the default settings. The package was used to demultiplex the data, generate FASTQ files, align the sequencing reads to the dm3 reference genome, filter cell and UMI (unique molecular identifier) barcodes.

### Filtering and Normalization

We used the cellranger Rkit to obtain matrices with cells in the columns and genes in the rows. For each condition (control and NSL1 knockdown) we retained only cells that had more than 2,000 nonzero genes. Next, we selected genes that had nonzero entries in more than 50% of the remaining cells. The filtering resulted in 3,258 cells for the control and 588 for the NSL1 knockdown and in total 1,787 genes.

We implemented a normalization technique, where we iteratively divide the matrix by the outer product between square root of the row and column means. This procedure converges quickly and results in a matrix, whose row means (= genes) and column means (= cells) equal unity. Here, we used 40 iterations.

For the PCA analysis, we concatenated the columns of the control and NSL1 knockdown and performed aforementioned normalization. The resulting balanced matrix was used as input to PCA and we plotted the rotation vectors for the first two principal components, showing that the second principal component is separating control and NSL1 knockdown cells.

To compare variation between conditions, we separately input the control and the NSL1 data. In both cases we obtained a balanced matrix, which we used to calculate the standard deviation for each gene. Note that the mean for each gene is fixed at one, such that the standard deviation for each row of the matrix is equal to the coefficient of variation, which is the standard deviation divided by the mean.

A confounding factor in the estimation of the coefficient of variation is the number of zero and nonzero elements per cell. To control for this factor, we subsampled the control cells by requiring that the distribution of non-zero genes is similar to the one found in NSL1 knockdown. We repeated subsampling 100 times and took the average gene coefficient of variation over these 100 subsamples.

scRNA-seq data was normalized using BASiCS (Vallejos et al. 2015). As input served unique molecular identifier counts for each gene and cell in the control and NSL1 knockdown experiment. To control for technical variability ERCC spike ins were used. We retained only cells that had more than 2,000 nonzero genes and had more than 10 ERCC spike in reads. The additional requirement of more than 10 ERCC spike in reads led to the reduction of the 588 cells to 560. Thereafter, we selected genes whose mean count was larger than 1 and ERCC spike ins that had at least one nonzero entry. Because there were many more control cells than NSL1 KD cells, we downsampled the control to the same number of cells as in the NSL1 knockdown (560). We repeated this 32 times. After running the BASiCS algorithm, we detected differential variability using the BASiCS_TestDE function. The assignment as a gene that changed variability required that all 32 subsamples flagged the gene as having an increased variability upon NSL1 KD.

The following datasets have been deposited in the Gene Expression Omnibus repository with the accession number GSE118726:

- MAPCAP – GST RNAi and NSL1 RNAi
- MNase- ChIP-seq with NSL1 and NURF301
- MNase seq – GST RNAi, NSL1 RNAi, INO80 RNAi and NURF301 RNAi
- Genomic DNA-IP-seq with GFP, NSL1, NSL3 and MCRS2
- Single cell RNA-seq – GST RNAi and NSL1 RNAi

**Supplemental references**

Chari S, Dworkin I. 2013. The conditional nature of genetic interactions: the consequences of wild-type backgrounds on mutational interactions in a genome-wide modifier screen. *PLoS Genet* **9**: e1003661.

Dobin A, Davis CA, Schlesinger F, Drenkow J, Zaleski C, Jha S, Batut P, Chaisson M, Gingeras TR. 2013. STAR: ultrafast universal RNA-seq aligner. *Bioinformatics* **29**: 15-21.

Dunn JG, Foo CK, Belletier NG, Gavis ER, Weissman JS. 2013. Ribosome profiling reveals pervasive and regulated stop codon readthrough in Drosophila melanogaster. *Elife* **2**: e01179.

Corona DFV, Längst G, Clapier CR, Bonte EJ, Ferrari S, Tamkun JW, Becker PB. 1999. ISWI Is an ATP-Dependent Nucleosome Remodeling Factor. *Molecular Cell* **3**: 239–245.

Deuring R, Fanti L, Armstrong JA, Sarte M, Papoulas O, Prestel M, Daubresse G, Verardo M, Moseley SL, Berloco M, et al. 2000. The ISWI Chromatin-Remodeling Protein Is Required for Gene Expression and the Maintenance of Higher Order Chromatin Structure In Vivo. *Molecular Cell* **5**: 355–365.

Flockhart I, Booker M, Kiger A, Boutros M, Armknecht S, Ramadan N, Richardson K, Xu A, Perrimon N, Mathey-Prevot B. 2006. FlyRNAi: the Drosophila RNAi screening center database. *Nucleic Acids Res* **34**: D489-494.

Gossett AJ, Lieb JD. 2008. DNA Immunoprecipitation (DIP) for the Determination of DNA-Binding Specificity. *CSH Protoc* **2008**: pdb prot4972.

Hu Y, Comjean A, Roesel C, Vinayagam A, Flockhart I, Zirin J, Perkins L, Perrimon N, Mohr SE. 2017. FlyRNAi.org-the database of the Drosophila RNAi screening center and transgenic RNAi project: 2017 update. *Nucleic Acids Res* **45**: D672-D678.

Kinkley S, Helmuth J, Polansky JK, Dunkel I, Gasparoni G, Frohler S, Chen W, Walter J, Hamann A, Chung HR. 2016. reChIP-seq reveals widespread bivalency of H3K4me3 and H3K27me3 in CD4(+) memory T cells. *Nat Commun* **7**: 12514.

Lam KC, Muhlpfordt F, Vaquerizas JM, Raja SJ, Holz H, Luscombe NM, Manke T, Akhtar A. 2012. The NSL complex regulates housekeeping genes in Drosophila. *PLoS Genet* **8**: e1002736.

Langmead B, Salzberg SL. 2012. Fast gapped-read alignment with Bowtie 2. *Nat Methods* **9**: 357-359.

Li H, Handsaker B, Wysoker A, Fennell T, Ruan J, Homer N, Marth G, Abecasis G, Durbin R, Genome Project Data Processing S. 2009. The Sequence Alignment/Map format and SAMtools. *Bioinformatics* **25**: 2078-2079.

Love MI, Huber W, Anders S. 2014. Moderated estimation of fold change and dispersion for RNA-seq data with DESeq2. *Genome Biol* **15**: 550.

Mendjan S, Taipale M, Kind J, Holz H, Gebhardt P, Schelder M, Vermeulen M, Buscaino A, Duncan K, Mueller J et al. 2006. Nuclear pore components are involved in the transcriptional regulation of dosage compensation in Drosophila. *Mol Cell* **21**: 811-823.

Perkins LA, Holderbaum L, Tao R, Hu Y, Sopko R, McCall K, Yang-Zhou D, Flockhart I, Binari R, Shim HS et al. 2015. The Transgenic RNAi Project at Harvard Medical School: Resources and Validation. *Genetics* **201**: 843-852.

Vallejos CA, Marioni JC, Richardson S. 2015. BASiCS: Bayesian Analysis of Single-Cell Sequencing Data. *PLoS Comput Biol* **11**: e1004333.

Yu L, Song Y, Wharton RP. 2010. E(nos)/CG4699 required for nanos function in the female germ line of Drosophila. *Genesis* **48**: 161-170.
